# A Catalogue of Machine Learning Algorithms for Healthcare Risk Predictions †

**DOI:** 10.3390/s22228615

**Published:** 2022-11-08

**Authors:** Argyro Mavrogiorgou, Athanasios Kiourtis, Spyridon Kleftakis, Konstantinos Mavrogiorgos, Nikolaos Zafeiropoulos, Dimosthenis Kyriazis

**Affiliations:** Department of Digital Systems, University of Piraeus, 185 34 Piraeus, Greece

**Keywords:** data analysis, machine learning, catalogue, supervised learning, prediction, healthcare

## Abstract

Extracting useful knowledge from proper data analysis is a very challenging task for efficient and timely decision-making. To achieve this, there exist a plethora of machine learning (ML) algorithms, while, especially in healthcare, this complexity increases due to the domain’s requirements for analytics-based risk predictions. This manuscript proposes a data analysis mechanism experimented in diverse healthcare scenarios, towards constructing a catalogue of the most efficient ML algorithms to be used depending on the healthcare scenario’s requirements and datasets, for efficiently predicting the onset of a disease. To this context, seven (7) different ML algorithms (Naïve Bayes, K-Nearest Neighbors, Decision Tree, Logistic Regression, Random Forest, Neural Networks, Stochastic Gradient Descent) have been executed on top of diverse healthcare scenarios (stroke, COVID-19, diabetes, breast cancer, kidney disease, heart failure). Based on a variety of performance metrics (accuracy, recall, precision, F1-score, specificity, confusion matrix), it has been identified that a sub-set of ML algorithms are more efficient for timely predictions under specific healthcare scenarios, and that is why the envisioned ML catalogue prioritizes the ML algorithms to be used, depending on the scenarios’ nature and needed metrics. Further evaluation must be performed considering additional scenarios, involving state-of-the-art techniques (e.g., cloud deployment, federated ML) for improving the mechanism’s efficiency.

## 1. Introduction

According to a recent survey [1], almost 60% of companies utilize machine learning (ML) techniques or artificial intelligence (AI) to support their decision-making processes. This percentage is expected to grow even more, highlighting the importance of the existence of mechanisms that can perform data analyses. The global AI market in 2021 was measured at 327.5 billion dollars, with companies such as IBM and Intel investing a great number of resources in ML and generally in AI to improve their products and services [2]. This suggests that dedicated mechanisms should exist for performing data analyses in collected data, including tasks like feature extraction and ML models’ training, applying those models to assess their accuracy, providing predictions, and strengthening their nature.

Such mechanisms are utilized in multiple domains and sectors, including transportation, finance, education, smart cities, and healthcare [3]. For instance, in the transportation domain those mechanisms are applied in route optimization, parking, streetlights, accident prevention/detection, and road anomalies [4]. In finance, they can be utilized for pattern recognition and financial econometrics [5], while in education they can be used for precision education [6]. Regarding smart cities, ML can be used for the effective utilization of unmanned aerial vehicles (UAVs) to assure the best services of 5G communications, as well as the energy-efficient utilization of smart grids (SGs) [7]. When it comes to healthcare, the use of ML is even more extensive due to the massive new opportunities that it can provide to this domain. Predicting the outcome of a patient’s disease, suggesting a suitable treatment, and creating an intervention or even a policy regarding public health are indicative examples of utilizing ML in this domain. An example of these opportunities can be the fact that considering traditional healthcare environments, the proper diagnosis of a disease depends on the healthcare practitioner’s decision, solely considering a patient’s symptoms. However, this may lead to unwanted errors that can result in increased medical costs, also affecting the quality of service provided to patients. Instead, expert healthcare systems that utilize data mining, ML, and AI techniques [1] could be used to emulate the decision-making ability of a human expert for answering simple questions like “What is the average age of patients who have heart disease?”, “Are there any female patients who are single and have been treated for heart diseases?”, and also complex ones like “Given the patient records, is it feasible to predict the probability of patients who diagnosed with a heart disease?”, “Is it possible to find the most significant risk factor that results to a heart disease?”. Considering the multidimensionality and heterogeneity of the health-related data that are hidden behind such questions, their efficient analysis can be managed by a variety of ML algorithms, thus leading to better decision-making [8]. Hence, making use of ML in healthcare can both reduce medical errors and decrease practice variation, while it can also assist the diagnosis’ process and improve the treatment plan [9]. Such a notion may be efficiently realized by identifying information in massive amounts of data by recognizing patterns and summarizing data into an understandable style. 

To get the most out of the provided data, the data analysis mechanisms should always ensure that they use the most suitable approach. Nowadays, a plethora of algorithms/techniques exist, each one being more suitable for specific use cases, thus making the selection of the proper one a real challenge. For instance, there may be cases of health incidents where a simple ML classification algorithm can be used to classify different patients based on their characteristics. There may also be other cases where a more complex ML prediction algorithm should be utilized to predict the onset of a disease. To this context, a variety of studies have been conducted to provide predictions either for diagnosis or for prognosis of different diseases and health conditions, comparing several ML algorithms and classifiers [2,3]. Most of those studies are use-case specific, not providing insights regarding the most suitable algorithm in diverse healthcare scenarios. What is needed is an on-demand real-time and ready-to-use catalogue of ML algorithms in the form of a list, indicating the proper ML algorithm that must be used under specific cases, considering real-time requirements and needs. Consequently, instead of randomly choosing an ML algorithm for predicting the cause or the onset of a disease that could possibly result in erroneous results and a time-consuming process, this ML algorithms’ catalogue could facilitate the overall choice and significantly improve the prediction process.

In this manuscript, a data analysis mechanism is proposed to deal with the abovementioned challenges, aiming to train different ML models on top of diverse healthcare-related datasets and use cases in order to derive the envisioned ML algorithms’ catalogue. As already stated, this catalogue will contain a list of the most proper ML algorithms to be used under specific healthcare scenarios, whereas through proper criteria selection these ML algorithms can be accordingly adapted and offered to their stakeholders. For instance, there may be cases where stroke prediction must be provided in real-time for a specific patient, and as a result Bernoulli Naive Bayes (BNB) should be performed. Real-time predictions are considered of crucial importance since there may exist several diseases, such as stroke, heart failure, or fainting episodes that may occur in unexpected cases, even in moments after abnormal bio-signals measurements have taken place [10]. As a result, if those abnormal measurements are detected on-the-fly and, based on an already trained ML healthcare disease model are classified as harmful, then the appropriate decisions could be made in real-time (e.g., real-time emergency alerts for hospitalization, contact of emergency services). However, in a different case, a stroke prevention policy should be provided, and as a result the Decision Tree would provide results of higher value. To achieve all these, the developed mechanism can collect data and perform various ML algorithms, such as BNB, K-Nearest Neighbors (KNN), Decision Tree, Logistic Regression, and Random Forest Classifiers to provide predictions regarding specific features that occur in healthcare data deriving from diverse scenarios. Based on the provided data and specific metrics that are generated after the training of the ML models, the mechanism selects the most proper algorithm to be used for predictions in similar future datasets and scenarios, under certain requirements. To evaluate such an approach, the mechanism exploits various heterogeneous datasets covering six different healthcare scenarios, ranging from stroke to COVID-19 data, among others, to verify its applicability in diverse healthcare scenarios and provide a complete ML catalogue based on an exploratory proof-of-concept. The major contributions of this manuscript lie not only on the ML catalogue itself, but also on the separate components that make up the whole proposed mechanism, which are further analyzed in the Sections below. More specifically, the first major contribution refers to the Gateway component that is responsible for the collection of the data, being capable of collecting both real-time and non-real-time data, regardless of their data format. Another major contribution refers to the Data Reliability component that is responsible for ensuring the reliability of the collected data, making use of several ML and natural language processing (NLP) techniques to efficiently complete its tasks. What is more, a notable contribution relies on the Model Training component, where a plethora of experiments are performed in order to conclude in the best suitable models’ parameters to be chosen for each different scenario, exploiting the hyperparameters tuning concept. To successfully capture and boost the reliability of the developed models, through the model evaluation component, the mechanism can estimate the overall performance of the trained ML algorithms by capturing a plethora of diverse metrics, referring to accuracy, precision, recall, F1-score, specificity, and confusion matrix for finally choosing the most proper algorithm to be applied on each given scenario, towards creating the proposed ML algorithms’ catalogue. Additionally, a notable contribution of the proposed mechanism refers to the Data Storage component that exploits the NoSQL databases concept in order to be able to efficiently handle and process the huge amounts of data that may be received. On top of the developed components, a major contribution of the proposed mechanism relies on the applied software architecture, which refers to the MicroServices Architecture (MSA). The latter provides a more efficient way for performing ML training and prediction than traditional architectures, such as the Monolithic Architecture (MA). 

The rest of this manuscript is organized as follows. Section 2 provides a literature review regarding the main principles of applying ML for predictions, mentioning how specific ML algorithms are utilized in the context of healthcare. Additionally, it depicts the overall architecture of the proposed mechanism, as well as the steps that have been followed towards the extraction of results. Section 3 states the derived results from the evaluation of the proposed mechanism, being followed by an extended analysis of them through proper graphs and charts. Section 4 provides a discussion of the captured results, also indicating the mechanisms’ limitations. Finally, Section 5 summarizes the manuscript’s overall work and provides insights for the following steps to tackle the faced challenges and limitations.

## 2. Materials and Methods

### 2.1. Machine Learning Principles and Algorithms

In recent years, ML has become a powerful tool in many fields of technology and other domains. It is devoted to creating algorithms and models that allow software applications to “learn” and perform accurate predictions upon the existing plethora of data. Thus, through ML, computers are capable of automatically improving their functionalities based on experience. To do so, computers need to utilize data derived from the real world so that they can “learn” from them and provide the needed predictions. To accomplish such a task, the applied ML techniques are usually separated into two categories, named supervised and unsupervised ML techniques. A labeled sample of training data is utilized in the first case to estimate/map the input data to the intended output. In the second case, no labeled data are supplied, and hence no specified intended result is provided. In every case, however, the goal remains the same, referring to the production of a ML model that can be exploited for classification, prediction, or any other related task. By the time that the model is generated, it is then imperative to be evaluated. To this context, the model’s performance can be assessed based on specific metrics, including accuracy, specificity, recall, precision F1-score, and confusion matrix, among others. 

In general, ML tools have grown in popularity in the healthcare area during the last few decades, where a variety of ML algorithms, including BNB, KNN, Decision Tree (DT), Logistic Regression (LR), Random Forest (RF), Neural Networks (NN), and Stochastic Gradient Descent (SGD) [10,11,12,13], among others, have been widely applied, aiming to detect key features of patients’ conditions, health risks, as well as diseases’ progression after treatment, exploiting information that derives from various complex medical datasets. Since a plethora of challenges and requirements exist that need to be faced regarding such concepts, various research approaches have been conducted towards this direction (i.e., generating ML models to provide predictions for diseases’ outcomes and assist in selecting the right treatment plan). Such approaches are further examined in Section 4 of the manuscript. Regarding the main challenges that need to be faced when utilizing ML in healthcare, those mainly refer to data collection, data management and, finally, data analysis. The latter aims to assist in precision medicine, which means creating the most proper model to result to the successful treatment protocol of patients based on their attributes and treatment context (e.g., provide the best treatment possible to patients suffering from diabetes). At the same time, since the volume of the underlying data is tremendous, all the corresponding ML approaches should make the most accurate predictions possible, without, however, exceeding certain computational limits. 

All of those challenges have been addressed by existing approaches that have been effectively applied in the medical research to construct prediction models towards correct decision-making [14,15], being use-case specific. As mentioned in [16], the selection of the best algorithm is based on the available dataset per use case and the diseases that those datasets refer to. Thus, in the context of this manuscript, for the validity and confirmation of the proposed mechanism’s produced results, a list of the most popular algorithms was selected (based on the current literature), having the ability to be applied concurrently in multiple datasets, since they are not tailored to be efficient solely for domain-specific datasets. Consequently, since the aim of this research is to cover multiple datasets from several healthcare scenarios, as well as to perform an unbiased performance evaluation based on different metrics and criteria, state-of-the-art algorithms were preferred to be used with a wider domain area application (e.g., healthcare, industry, manufacturing), avoiding already known algorithms for their high-performance explicitly in the medical domain (e.g., XGBoost, AdaBoost, GDBT). Furthermore, it should be noted that although the latter algorithms (i.e., XGBoost, AdaBoost, GDBT) may be more efficient in certain medical domains, the current literature shows that their applicability and efficiency have not been experimented and evaluated as a whole in such a wide area of healthcare scenarios, such as the ones that are investigated in the current manuscript. Although isolated cases exist, such as AdaBoost, which has been evaluated on top of eczema datasets [17], XGBoost, which has been experimented considering asthmatic patients [18], type-2 diabetic patients [19], GDBT that has been put into practice upon predicting patients’ care needs [20], and Parkinson’s disease [21]. However, to the best of our knowledge, none of the aforementioned research works evaluate these algorithms on top of the same use cases simultaneously. As a result, their prior performance and applicability would be equally unknown, as in the case of using the current research algorithms (e.g., Naïve Bayes, KNN). For this reason, the proposed mechanism utilizes seven widely used and well-established ML algorithms, namely the BNB, KNN, DT, RF, LR, NN, and SGD, to train models to perform predictions across diverse healthcare anomalies’ scenarios (e.g., diabetes, heart failure, stroke, COVID-19, breast cancer, and chronic kidney disease). Based on specific metrics (i.e., accuracy, recall, precision, F1-score, specificity, and confusion matrix), the proposed mechanism compares the algorithms’ efficiency and chooses the most proper one to be applied on each given scenario, towards creating the proposed ML algorithms’ catalogue. The basic principles of all the exploited algorithms are further depicted in the following sub-sections.

#### 2.1.1. Bernoulli Naïve Bayes (BNB)

**Figure 1:**
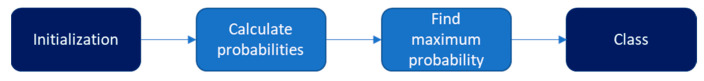
Indicative example of BNB steps.

BNB is based on Bayes’ theorem for finding the conditional occurrence probability of two events based on the occurrence probabilities of each event. When the class variable is supplied, BNB considers the presence/absence of a certain attribute of a class to be unconnected to the presence/absence of any other attribute, while the features are independent binary variables that reflect whether a condition will occur or not [22]. BNB’s model can be created as shown in Formula (1):

(1) $${P(x}_{i}\;|\;y)=P(i\;|\;y)x_{i}+\left(1-P(i\;|\;y\right))\left(1-x_{i}\right)$$

where ${P(x}_{i}\;|\;y)$ is the probability of $x_{i}$ given that evidence y has already occurred, and y can have only values 0 or 1. The needed steps to apply BNB are shown in Figure 1.

Based on the literature, the BNB classifier is used in a variety of health-related use cases, which are highlighted below through a multitude of research case studies. More specifically, in [23], the authors created a decision support system based on BNB to predict heart failure disease. In a similar study [24], the authors used BNB to predict heart disease, resulting into a greater success rate of correct prediction than previous approaches. Another important use of BNB in health-related sectors is liver disorders. To predict three major liver disorders using their different characteristics [25], the authors also employed BNB. By comparing BNB with NB, they discovered that NB outperforms its counterpart in predicting the three liver diseases of liver cancer, cirrhosis, and hepatitis. In [26], the authors created a human–machine semi-automated system based on BNB to classify injury tales using a huge administrative database, discovering that the created method had a very high overall accuracy. In another study [27], to identify illnesses, the authors created a patient-centered clinical decision support system employing BNB and cloud computing technologies. A comprehensive overview of the use of BNB in medical data mining is also discussed in [28]. Most recently, in [29], the authors performed an analysis based on the symptoms observed in people affected by COVID-19, and among other algorithms they used BNB to perform classification to find the best performance metrics.

#### 2.1.2. K-Nearest Neighbors (KNN)

**Figure 2:**
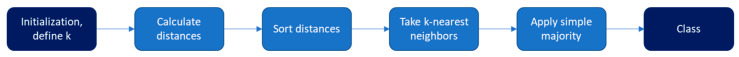
Indicative example of KNN steps.

KNN is a non-parametric algorithm that is widely used for classification, getting as an input the k closest training instances in a dataset, and producing as an output the class of a requested instance, where the majority votes of the instance’s neighbors exist [30]. KNN’s model can be created following Formula (2):

(2) $${\left(\overset{n}{\underset{i=1}{\sum }}{\left|x_{i}-y_{i}\right|}^{P}\right)}^{\frac{1}{P}}$$

where *p* > 0 and $x_{i}\;,$ $y_{i}$ are the vectors to find the similarity distance between the x and y instances. The steps for applying KNN are summarized in Figure 2.

According to the literature, KNN is used in a variety of health-related fields. To be more specific, for stroke prediction, the authors in [31] outlined that the accuracy rate achieved from KNN is very high, whereas in the same notion the authors in [32] stated that KNN scores the highest percentage of accuracy against the other algorithms that are exploited for predicting healing of COVID-19 patients. In addition, in [33], a conceptual framework for developing sustainable digital innovation hubs was examined on top of different algorithms performed on diabetes data, where it was observed that KNN scores were lower in terms of efficiency. However, for cases of kidney disease prediction, the authors in [34] noted that KNN has increased levels of accuracy. In [35], a correlation analysis for determining effective data in ML was performed on top of heart failure datasets, proving that KNN scores the highest accuracy rate. In [36], the authors used KNN to classify arrhythmia beats, while in [37] the authors efficiently predicted heart disease using KNN. Another study [38] compares the performance of KNN and BNB on thyroid detection, whereas most recently, the research in [30] performs a comparison of the performance of KNN and its variations for disease prediction.

#### 2.1.3. Decision Tree (DT)

**Figure 3:**
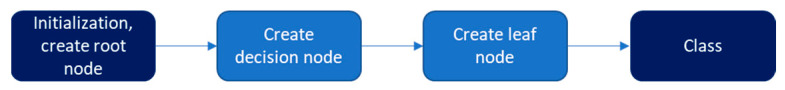
Indicative example of DT steps.

DT is a predictive modelling approach used in statistics, and ML is one of the most popular approaches for producing classifiers [39]. It is analogous to the flowchart structure, in which each internal node represents a condition on an attribute, each branch represents the condition’s outcome, each leaf node represents the class label, and the final choice is made after computing all the attributes [40]. In essence, it employs a decision tree to move from observations of an item (represented by branches) to inferences about the target value of the item (represented by leaves) [41], formulated by applying Formula (3):

(3) $$G=\overset{C}{\underset{i=1}{\sum }}p\left(i\right)*\left(1-p\left(i\right)\right)$$

where C is the total number of features and p(i) is the probability of picking the data point with feature i. The steps for applying DT are shown in Figure 3.

According to a comparative analysis of ML classifiers for stroke predictions [42], it is observed that the accuracy rate noted for DT is of high value, whereas in [43], COVID-19 mortality levels are predicted following the DT algorithm scores, which were not satisfactory enough. In addition, for diabetes prediction, a comparative study in [44] demonstrates that DT achieves results of high score, while considering the cases of predicting and diagnosing breast cancer and kidney disease. In [45,46], DT is selected as an effective option of classification algorithms, along with recursive feature elimination techniques, resulting into high accuracy rates. Additionally, in [47], in the context of heart failure data, it has been noticed that DT scores a medium level of accuracy rate, among a list of ML algorithms that are applied for multiple diseases’ prediction.

#### 2.1.4. Random Forest (RF)

**Figure 4:**
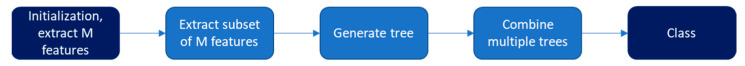
Indicative example of RF steps.

RF is an ensemble learning approach widely used for classification, which works by generating a large number of decision trees during training. It is similar to DT, except the fact that the algorithm builds a forest of decision trees with attribute sites picked at random. It has the benefit of increased computer efficiency, which improves forecast accuracy without significantly increasing the computational cost. It is also capable of predicting up to hundreds of explanatory factors [40], whilst it can be formulated as depicted in (4):

(4) $$Gini\;Index=1-\overset{n}{\underset{i=1}{\sum }}{\left(P_{i}\right)}^{2}=1-\left[{\left(P_{+}\right)}^{2}+{\left(P_{-}\right)}^{2}\right]$$

where $P_{+}$ is the probability of a positive feature and $P_{-}$ is the probability of a negative feature. The steps that need to be followed to apply RF are summarized in Figure 4.

In the medical profession, RF is frequently used to pick the proper and ideal mix of symptoms and illness components. This is accomplished by reviewing the patient’s medical records [48]. In accordance with the literature on predicting stroke outcomes, it is observed that in [49] the accuracy rate noted for the RF algorithm is of high value, whereas in [50], a computational intelligence-based model is used for mortality rate prediction in COVID-19 patients, proving that RF scores a high-value accuracy rate. In addition, on a diabetes prediction using ML algorithms with feature selection and dimensionality reduction, a comparative study was done in [51], where it is observed that RF achieves a medium-value score of accuracy. Additionally, regarding predicting breast cancer biopsy outcomes in [52] and developing an insulin resistance model in the context of the kidney disease in [53], both studies show that RF has high values of accuracy rates. Additionally, regarding using effective data mining techniques for heart failure, in [54] the authors noticed that RF is among the best candidates of ML algorithms, with scores of high accuracy.

#### 2.1.5. Logistic Regression (LR)

**Figure 5:**
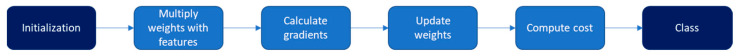
Indicative example of LR steps.

In its most basic form, LR is a statistical model that employs a logistic function to represent a binary dependent variable. In essence, it divides training data into two categories (“0” and “1”), which relate to the Bernoulli distribution [55], while when there are more than two possible outcomes, multinomial logistic regression is used. In general, LR is used in regression analysis to estimate the parameters of a logistic model (a form of binary regression), as depicted in Formula (5):

(5) $$p\left(x\right)=\frac{1}{1+e^{-\left(\beta_{0}+\beta_{1}x\right)}}$$

where $\beta_{0}$ = −μ/s and $\beta_{1}$ = 1/s. The steps that need to be followed to apply LR are summarized in Figure 5.

The existing research reveals that LR is used in a variety of health-related fields. Study [56] predicts the risk of drug intoxication mortality using LR, whereas the study in [57] uses LR for pancreatic cancer classification. Most recently, ref. [58] performed an evaluation of brucellosis risk variables in dairy cattle using LR and classification trees. Moreover, on the stroke usage scenario, it is observed that in [59] where the authors try to predict the motor function in stroke patients, the accuracy rate that is noted for LR is not of high efficiency. Additionally, regarding the mortality rate prediction of COVID-19 patients, in [50] it is observed that LR scores a high accuracy rate, whereas in [60], considering a comparison of ML algorithms for diabetes prediction, it is observed that LR achieves a high score for splitting method. Additionally, regarding breast cancer prediction in [45] and the study of predicting kidney disease in [61], among several algorithms that have been compared, it has been identified that LR has high accuracy rates. Additionally, in [54] through research for predicting heart failures, it was noticed that LR scores medium levels of accuracy rate.

#### 2.1.6. Neural Networks (NN)

NN, also known as artificial neural networks (ANNs), are a sub-set of ML inspired by the human brain, mimicking the way that biological neurons signal to one another. A widely known NN learning algorithm, among many others, is multi-layer perceptron (MLP), which is an extension of the least mean squared rule [62]. MLP learns a function f(.): R_m_ → R_o_, where _m_ is the number of input dimensions and _o_ is the number of output dimensions. Given a set of features x and a target, MLP can learn a nonlinear function approximator for classification. It differs from LR in the sense that one or more non-linear levels, known as hidden layers, can exist between the input and the output layers. One buried layer with scalar output exists, whereas the leftmost layer (i.e., input layer) is made up of neurons that represent the input characteristics. Each neuron in the hidden layer performs a weighted linear summation on the preceding layer’s values, while the last hidden layer values are received by the output layer and transformed into output values [63].

**Figure 6:**
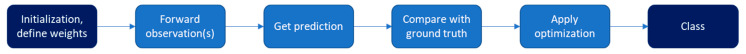
Indicative example of MLP steps.

The steps that need to be followed to apply MLP are summarized in Figure 6.

In the medical field, there is a growing trend of NNs being used to learn about fault tolerance, generalization, and the environment in medical diagnosis, as stated in [44], whereas by exploiting data mining techniques in [54] and NNs, it is observed that high levels of accuracy are achieved. More deeply emphasizing in MLP, a study [64] used MLP for breast cancer classification. Additionally, in [65], the authors performed cardiac arrythmia classification using MLP. Most recently, a study [66] presented a medical data MLP classification approach based on a biogeography-based optimization algorithm with a probability distribution. Moreover, according to the literature on stroke prediction from electrocardiograms by deep neural network, the results in [67] show high accuracy values. A reliable neural network-based tool for predicting confirmed cases, recovered cases, and deaths from COVID-19 has proved to be very useful to health advisors to take appropriate measures to control the epidemic [68]. A realistic framework has been proposed for predicting the diagnosis of diabetes in [69], resulting into high-value results, while in [70], the authors reviewed various approaches for breast cancer diagnosis and compare MLP and convolutional NN methods, concluding with the production of quite reliable results in both cases.

#### 2.1.7. Stochastic Gradient Descent (SGD)

**Figure 7:**
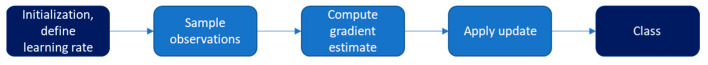
Indicative example of SGD steps.

In gradient descent (GD), a cost function is explicitly minimized by several neural network learning techniques. Backpropagation, for example, employs GD to minimize the mean squared error criterion by modifying the weights after each sweep over the training dataset in the whole GD process. SGD, on the other hand, has been shown to be quicker, more reliable, and less prone to reaching undesirable local minima than GD. The weights in this approach are adjusted after each example is shown based on the gradient of the loss function [71]. The applied formula for such a process is depicted in Formulas (6) and (7):

(6) $$\underset{a\;\to 0}{\lim}L\left(x+au\right)=u^{T}\;\nabla_{x}L\left(x\right)$$

(7) $$u^{T}\;\nabla_{x}L\left(x\right)=u\;\nabla_{x}L\left(x\right)\;cos\theta$$

where x is assumed to be a single vector, and u denotes the unit vector or direction in which x should ideally be altered, aiming to find a u such that $u^{T}\;\nabla_{x}L\left(x\right)$ is minimized [72]. The steps that need to be followed to apply SGD are summarized in Figure 7.

The research suggests that SGD may be used in a variety of medical use cases, such as investigating the primary causes of a disease or forecasting the likelihood of illness development based on the factors impacting the condition [73]. What is more, in [74], an ensemble framework for improving brain stroke prediction performance is used along with SGD, resulting into high accuracy results. Regarding COVID-19, different X-rays are compared through SGD, with the algorithm scoring a relatively moderate result in the accuracy coefficient in [75]. Algorithms such as SGD also overcome performance issues and speed up convergence, especially on large datasets as provided in [76], whereas considering breast cancer prediction, it is observed that according to [77], SGD scores a relatively low accuracy rate. Finally, according to a performance analysis of SGD on top of kidney disease datasets, in [78] it is observed that SGD has high accuracy rates, while for improving the prediction of heart failure patients, SGD has been proved not to be reliable enough, with moderate levels of accuracy rate in [54].

### 2.2. Proposed Machine Learning Approach

**Figure 8:**
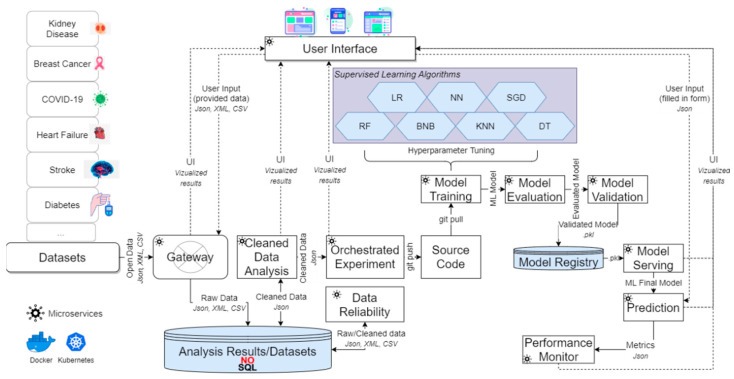
Overall mechanism architecture.

As already stated, there exist a plethora of ML algorithms, each one of them being functional and effective at different contexts and levels for performing efficient and trustworthy predictions. Especially into the context of healthcare, where timely predictions are considered of crucial importance, this manuscript aims to identify the most suitable ML algorithm to be triggered on top of different healthcare-related scenarios, considering the domain of the ingested datasets, as well as a set of criteria such as results’ accuracy, precision, and specificity, among others. Based on that, the mechanism depicted in Figure 8 will ingest datasets covering different scenarios (e.g., diabetes, heart failure, stroke, COVID-19, breast cancer, kidney disease) and will identify the most suitable ML algorithm to be used for each separate case to predict the onset of a disease more efficiently.

To achieve the abovementioned task, the mechanism relies on a Kubernetes cluster to easily scale the proposed application compared to virtual machines and speed up the overall delivery process. This cluster consists of various microservices [79], each one of them being hosted in a docker container and being responsible to perform a specific functionality (e.g., data ingestion, data cleaning, Model Training). 

In deeper detail, as shown in Figure 8, the whole process begins by collecting the required datasets to be analyzed. Thus, through the provided Gateway microservice, all the data are retrieved by the corresponding data sources. To this end, it should be noted that the Gateway microservice can collect either streaming or non-streaming data from external sources through dedicated application programming interfaces (APIs) that have been developed, regardless of their size and format [80,81]. The data are then converted to JavaScript Object Notation (JSON) format, and are stored in a NoSQL database (i.e., MongoDB) in the form of collections [79]. At this point, it is worth mentioning that every microservice that processes the aforementioned data, such as the Data Reliability microservice that will be analyzed later on, will also utilize the data in their JSON format. As soon as the data are stored into the database, the Data Reliability microservice is applied. The latter is responsible for automatically preprocessing and cleaning all the collected stored raw data by performing cleaning operations for identifying and rapidly removing duplicate records, missing values, outliers, and syntactic errors that may exist into the data. To perform such task, the Data Reliability microservice performs data cleaning based on a set of constraints that are part of certain structures called schemas, which each dataset’s features should satisfy (i.e., rule-based data cleaning). Those constraints/schemas are generated either by dataset experts or automatically, with the use of natural language processing (NLP). More specifically, NLP is utilized in the scope of the Data Reliability microservice as a way to compare a newly uploaded dataset (i.e., whose corresponding data schema is not available) to data schemas that were previously generated by the microservice, thus minimizing the computational cost of the whole data cleaning process. Every feature of the new dataset is compared to the features of the old datasets regarding the syntactic and the semantic similarity of the features, as well as the characteristics of the features. The computation of the syntactic similarity takes place by utilizing two commonly used techniques for accomplishing such task, namely the Jaccard similarity [82] and the Cosine similarity [83]. Regarding the semantic similarity, a more complex technique is used that is based on Transformers, which are types of Deep Learning (DL) models that differentially weight the input data [84]. If, by utilizing those NLP techniques, the Data Reliability microservice succeeds in finding a high similarity (syntactic and semantic) between the new feature and an old feature (exceeding the set threshold of 70% based on previous research [85]), then the corresponding rule of the old feature is used to validate the new feature, thus eliminating the need for generating a brand-new rule (or even a data schema). In the case that the similarity of the new feature to an old one is not satisfactory, then either the rules of the most similar feature are selected, or a data expert should intervene to create new rules for this specific feature/dataset. Additionally, in the cases where missing values are identified based on the set/found constraints, the Data Reliability microservice utilizes ML algorithms to predict the missing values. To be more specific, the mechanism firstly detects such values by performing several SQL-like queries on the data (e.g., the mechanism may SELECT all the attributes WHERE their value is not following a specific pattern), and then utilizes the KNN algorithm to predict the missing values based on other attributes’ behavior, nature, or similar patterns. As a final step, the mechanism replaces those missing values with the predicted ones, again following several SQL-like queries on the data (e.g., the mechanism may UPDATE an attribute WHERE a specific anomaly was detected). To this end, it must be noted that the mechanism can also delete the value of an unpredicted attribute following the notion of SQL-like queries (e.g., the mechanism may DELETE an attribute WHERE its value could not be predicted). It is clear that the latter functionality could be efficiently replaced through simple SQL queries, but in the proposed mechanism’s case, it was best chosen to follow the SQL queries nature and increase their complexity with ML methods to more efficiently address a wider area of missing values. For instance, through using simple SQL queries, the values that could be replaced by pattern mining techniques (i.e., a result of ML) would not be efficiently predicted, leading possibly to the overall deletion of entire dataset rows, which could affect the overall analytics’ outcomes and the gained insights, since a smaller dataset would be used in the end for the overall ML Model Training [86]. This could have major effects for decision-makers, especially considering the nature of the healthcare domain that requires high volumes of reliable data for high-value outcomes. By the time that the data cleaning process gets completed, the Data Reliability microservice stores the cleaned data values in a new collection in the database.

As soon as the collected data are effectively preprocessed, the Data Analytics microservices are implemented. Specifically, the cleaned data analysis microservice applies exploratory data analysis (EDA) as an approach for analyzing cleaned datasets to summarize their main features. EDA is an approach that looks at the data from as many angles as possible, always on the lookout for finding some interesting features [87]. EDA’s goal is to analyze datasets to summarize their main characteristics, often using statistical graphics and other data visualization methods. Then, the Orchestrated Experiment microservice is applied. The current most popular experimental methodology in ML for accomplishing such a task is to firstly come up with a hypothesis about the algorithms under investigation, then perform experiments explicitly designed to test this hypothesis, and finally interpret the produced results. Every parameter or value in every hypothesis made by the proposed mechanism is stored into a git source repository, from where it is then available to repeat the experiment, at the same time being available for the rest of the microservices. Essentially, the Orchestrated Experiment microservice is the parent microservice of the training, evaluation, and validation of the mechanism’s ML models’ microservices. Initially, the Orchestrated Experiment microservice prepares the data for the ML models, by randomly splitting 80/10/10 the data into training, validation, and test sets. In sequel, data transformations (i.e., the process of changing the format, structure, or values of the data) and feature engineering (i.e., the process of selecting, manipulating, and transforming the underlying data into features that can be used in supervised learning) are applied as an approach of analyzing the cleaned datasets to summarize their main features. As soon as this process gets complete, the training of the mechanism’s ML models occurs through the Model Training microservice. Regarding the algorithms that are used to train the mechanism’s models, as mentioned in Section 2.1, these refer to: (i) BNB, (ii) KNN, (iii) DT, (iv) LR, (v) RF, (vi) NN, and (vii) SGD, where all of the chosen algorithms include various hyperparameters that must be tweaked before the algorithms are ready to be executed (further analyzed in Tables 8–14). Simply said, parameters in ML refer to the values that a learning algorithm may independently alter as it learns, and these values are influenced by the hyperparameters that the data analyst specifies. Therefore, before training begins, hyperparameters are specified, and the learning algorithm utilizes the set hyperparameters to learn the parameters. Parameters are constantly modified behind the scenes throughout training, and the final ones derived by the finalization of the training compose the final model. As a result, selecting the appropriate hyperparameter values is critical since it has a direct influence on a model’s performance when employed during Model Training. Hence, in the context of the proposed mechanism hyperparameters, tuning is performing all the abovementioned process in order to determine the structure of each ML algorithm and how the algorithm is taught. The initial settings of each algorithm’s hyperparameters are the default values stated in the utilized software packages that are based on recommendations/past research [88,22]. 

After the training phase, each model is evaluated and validated with the Model Evaluation and Model Validation microservices. More specifically, to identify the best suitable training algorithm and make the needed prediction upon the given data, during the training phase, the mechanism estimates the metrics of (i) classification accuracy that captures the number of right guesses divided by the total number of predictions, (ii) F1-score (Formula (8)) that combines the metrics of precision (Formula (9)) and recall (Formula (10)), and (iii) specificity (Formula (11)) that refers to the likelihood of a negative test, assuming that the test is genuinely negative.

(8) $$F1\;\mathrm{Score}=2\times \frac{\mathrm{Precision}\;\times \mathrm{Recall}}{\mathrm{Precision}+\mathrm{Recall}}$$

(9) $$\mathrm{Precision}=\frac{\mathrm{True}\;\mathrm{Positives}}{\mathrm{True}\;\mathrm{Positives}+\mathrm{False}\;\mathrm{Positives}}$$

(10) $$\mathrm{Recall}=\frac{\mathrm{True}\;\mathrm{Positives}}{\mathrm{True}\;\mathrm{Positives}+\mathrm{False}\;\mathrm{Negatives}}$$

(11) $$\mathrm{Specificity}=\frac{\mathrm{True}\;\mathrm{Negatives}}{\mathrm{True}\;\mathrm{Negatives}+\mathrm{False}\;\mathrm{Positives}}$$

Additionally, the train–validation–test scores are used for each dataset that is divided into training set, validation set and test set. The purpose of this analysis is to prove the reason why an algorithm has been chosen, compared to the other cases. The training set is used to train the model and teach it about the hidden features/patterns in the data. The training set should include a broad range of inputs so that the model may be trained in a variety of circumstances and forecast any previously unknown data sample that may arise in the future. As for the validation set, the basic reason behind separating the dataset into a validation set is to keep the model from overfitting, which occurs when the model gets proficient at categorizing samples in the training set but is unable to generalize the results and make accurate classifications on data that it has not seen before. Finally, the test set is a different collection of data used to validate the model once it has been trained. In terms of accuracy and other measures, it delivers an impartial final model performance metric. Therefore, it is desirable to observe a high percentage in all the three targets, whereas the deviation rates should be around 1%–2%.

**Figure 9:**
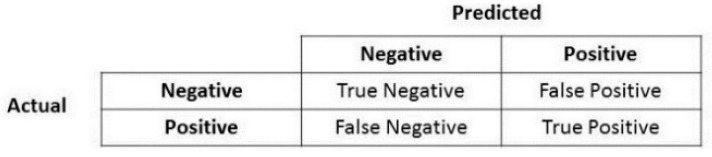
Confusion matrix.

In combination with the above, another important element in the algorithms’ performance in the field of ML, and in particular in the problem of statistical classification, is the confusion matrix. The main concepts of a confusion matrix, include: (i) True positive (TP) values for cases that the prediction is realistically positive, (ii) True negatives (TN) values for cases that the prediction is realistically negative, (iii) False positives (FP) values for cases that the prediction is positive but not realistic, (also known as “type I error”), and (iv) False negatives (FN) values for cases that the prediction is negative but not realistic, (also known as “type II error”). This matrix (Figure 9) contains two rows and two columns and uses the number of TP, TN, FP, and FN. Each column represents the number of values in their categorized class, whereas each row denotes the number of items in their real class. The correct predictions are on the diagonal of the matrix, while the remaining cells show the incorrect predictions. Ideally, it is preferrable for the diagonal to contain large numbers, while the rest of the cells to tend to be zero as much as possible. Hence, the best algorithm is the one that has the most elements in the main diagonal in the matrix. 

As soon as all these metrics are captured for each trained algorithm, the trained models are stored in the Model Registry (i.e., a centralized repository where model developers may submit production-ready models for easy access) in the form of pickle files (i.e., .pkl file extension), and then are retrieved by the Model Serving microservice (i.e., a model serving that hosts ML models and makes their functions available via APIs so that other microservices can incorporate into their systems) in order to serve the Prediction microservice. In essence, the Prediction microservice is the final stage in the proposed mechanism, which tries to warn the physicians and the caregivers of the likelihood of events and outcomes before they occur, assisting them in preventing and curing health concerns to the greatest extent feasible.

On top of this process, the mechanism provides a suitable user interface (UI) to provide the ability to its users (i.e., researchers, data scientists, clinicians, physicians, caregivers) to insert new patient data, by uploading them with a simple drag and drop functionality in a specific form. Through this UI, data scientists and researchers can perform data analysis and ML experiments with a plethora of heterogeneous datasets. Through this way, new ML models are served and are available for predictions for all the potential users. Finally, the results and the performance metrics of each algorithm are monitored through the Performance Monitor microservice of operations. These metrics help the mechanism to determine how the deployed model is performing from a usage point of view. These metrics include: (i) throughput for calling the Prediction microservice (i.e., number of requests), (ii) latency when calling the Prediction microservice (i.e., average response time), (iii) IO/Memory/CPU usage when performing prediction (i.e., average consumption), (iv) disk utilization (i.e., average consumption), and (v) system uptime, whereas all of these metrics should be calculated over different time frames, as desired.

By successfully accomplishing all the abovementioned steps, the mechanism achieves the uploading and analysis of various health data, providing the appropriate visualization and interpretation of the results of predicting the occurrence of a disease. To this end, it should be noted that the difference with other data visualization and analysis tools, such as Weka [89] and AutoML [90], is the fact that the proposed mechanism is based on a specific domain, that of healthcare, and not on a general visualization approach. That is why specific emphasis has been provided into domain-specific sub-mechanisms in the form of microservices—to prioritize and more efficiently collect, enhance, analyze, and visualize the ingested data. This is in contrast with AutoML techniques and the Weka software, as on the one hand they are more applicable into a wider area of domains, but on the other hand they are not sufficiently considering the data requirements of the healthcare domain (e.g., data prioritization issues, data confidentiality, data provenance logging), which are tackled from the current research’s mechanism. Additionally, the downside of AutoML is its lack of business intuition. Despite AutoML leading to a production-ready model more quickly, it would not provide justification regarding the use of ML or an interpretation of the prediction results, let alone the selection of a specified problem to attempt to solve from the multitude of tools available. Furthermore, Weka as a software has a less active community, and is efficient only in the cases where data are well cleaned and prepared, while based on current research, it has a large inability of handling large amounts of data. Moreover, considering its outdated UI, it is also unable to follow current researchers’ requirements for enhanced visualizations and better explainability results. Hence, considering the aforementioned drawbacks, the aim of this mechanism is to go beyond their shortcomings and provide an end-to-end architecture for healthcare data handling, analysis, and outcomes’ interpretation and visualization.

## 3. Results

### 3.1. Datasets Description

The applicability of the proposed mechanism was measured by evaluating all the different ML algorithms on six diverse datasets selected from various available data repositories, referring to different health anomalies and scenarios (i.e., diabetes [91], stroke [92], heart failure [93], COVID-19 [94], breast cancer [95], kidney disease [96]). More specifically, diabetes data were chosen because diabetes refers to a set of metabolic disorders from which millions of people suffer worldwide and, as a result, it is imperative to find the best prediction model to avoid implications and provide the best treatment possible. In the case of the stroke-related dataset, as well as the heart-failure-related dataset, those were chosen since both stroke and heart failure events occur regularly and are fatal in about 20% of the cases [97]. COVID-19 data were chosen because the COVID-19 pandemic radically changed the ordinary way of life and ML models are needed to predict the short-term (i.e., Intensive Care Units (ICU) visits) and long-term implications of this disease (i.e., other medical conditions such as heart failure). Breast cancer data were used because breast cancer is one of the most common types of cancer in women, and ML models could help physicians to determine whether a tumor is malignant or not. Finally, kidney disease is also a chronic disease for which, even though it mostly affects older people, its symptoms may occur in an earlier stage in life. To predict the occurrence of kidney disease, ML models can also be utilized, and that is why a kidney disease-related dataset was also used in this study.

The selection of the aforementioned diseases, apart from the COVID-19 case, regards the selection of some of the major chronic diseases in which patients are subject to multiple drug regimens. More specifically, two of the four main categories of chronic non-communicable diseases as indicated by WHO [98] have been selected, referring to cardiovascular diseases (stroke, heart failure) and diabetes mellitus (diabetes). In addition to these categories, there are also breast cancer and kidney disease, which do not belong to any of the four categories but belong to the wide range of chronic diseases. In addition, the choice of the COVID-19 case is due to the fact that it is a recent healthcare topic studied by the entire research community to interpret its possible linking with chronic diseases. At this point, it is necessary to clarify that chronic diseases share common factors (common characteristics) and risk situations. While some risk factors, such as age and sex, cannot be changed, many behavioral risk factors can be changed, as well as several intermediate biological factors such as high blood pressure or body mass index. Additionally, economic (e.g., working status) and physical conditions (e.g., place of residence) influence and shape behavior and indirectly influence other biological factors (e.g., single or married, smoker or non-smoker). Identification of these common risk factors and conditions is the conceptual basis for a comprehensive approach to any chronic disease being studied. Equally important is the contribution of laboratory data, such as red or white blood cells and other data, which help clinicians to provide a better picture of the control and prevention of potential patients’ health-related risks.

In further detail, the diabetes dataset spans ten years (1999–2008) of clinical treatment across 130 US hospitals and integrated delivery networks, including more than 50 variables characterizing patient and hospital outcomes. However, for the current experimentation, the main features that were selected were race, gender, age, admission type, discharge disposition, admission source, time in hospital, number of procedures, number of medications, number of inpatient visits, number of diagnoses, glucose serum test result, A1c test result, and change of medications (Table 1). The characteristics were chosen because they reflect the main features of a patient’s background and main clinical features.

Regarding the stroke event dataset, it consists of clinical features for predicting stroke events, like gender, age, and various diseases. Indicatively, some of the chosen features of the above dataset were gender, age, hypertension, heart disease, and smoking status (Table 2). The selected features were considered necessary for the final prediction result, because in the case that a patient has hypertension or not, it is a feature with significant role. Knowing whether the patient is married or not, whether he/she lives in a city or the suburbs, as well as his/her occupation, also are of great importance. Equally important are the body mass index metrics or glucose levels, as well as smoking category.

As for the heart failure dataset, it is made up of 299 heart failure patients’ medical records obtained from the Faisalabad Institute of Cardiology. The patients ranged in the age from 40 to 95 years old and included 105 women and 194 men. For this dataset, the following features were selected for the experimentation: age, anemia, creati-nine_phosphokinase, diabetes, and ejection_fraction (Table 3). The features reported to make the ML models are general patient targets, such as age, gender, and smoking habits. Additionally, there included data from clinical laboratory tests that are considered necessary metrics for the state of a patient’s indicators.

As for the COVID-19 dataset, ML models were created exploiting train-test split techniques using 18 laboratory data features from 600 individuals. Hence, for this dataset, the following features were selected: age quantile, hematocrit, hemoglobin, platelets, and red blood cells (Table 4). Predicting whether a patient ends up with pneumonia or not due to COVID-19 implications requires the patient’s laboratory data, because the laboratory indicators are quite important for making new decisions from health professionals.

Breast cancer is the most frequent malignancy in women worldwide. It is responsible for 25% of all cancer incidences and afflicted approximately 2.1 million individuals in 2015. The main obstacle to its detection is determining whether tumors are malignant (cancerous) or benign (non-cancerous). The chosen characteristics are included in Table 5, which are quite important for the broad picture of the patient, and thus for training the models in order to produce the most accurate outcome predictions. 

Finally, the last of the datasets is that of kidney disease. The corresponding data for this scenario were collected from a competition of the electronic repository Kaggle, with the amount of this data reaching 399 patient records with the control of various laboratory elements deemed necessary for training the models and safely making decisions about kidney disease predictions. The characteristics that were selected for investigating whether there is a risk of developing kidney disease or not are related to age, blood pressure, specific gravity, albumin, etc. (Table 6).

### 3.2. Evaluation Environment

The source code of every microservice of the proposed mechanism has been implemented in Python language [57] exploiting the Flask framework (v.2.0.1) [58]. For the data analysis the Pandas framework [63] was exploited, for the ML algorithms the scikit-learn libraries [64] were used, whereas the UI was implemented using Angular [71]. Docker [64] was used for containerizing the microservices, whilst Kubernetes [65] in a minikube [66] on a centos7 [87] server was exploited for orchestrating the Docker containers. The development of all the above is accomplished by an 8-core processor with 32 GB of memory and has a centos7 as an operating system. Apache JMeter [99] was used as a testing tool for capturing the algorithms’ training time metrics. Finally, batch learning [100] was used throughout every experiment. Concerning the results of the mechanism, these are depicted below, following step-by-step the process explained in Section 2.2.

### 3.3. Evaluation Results

**Figure 10:**
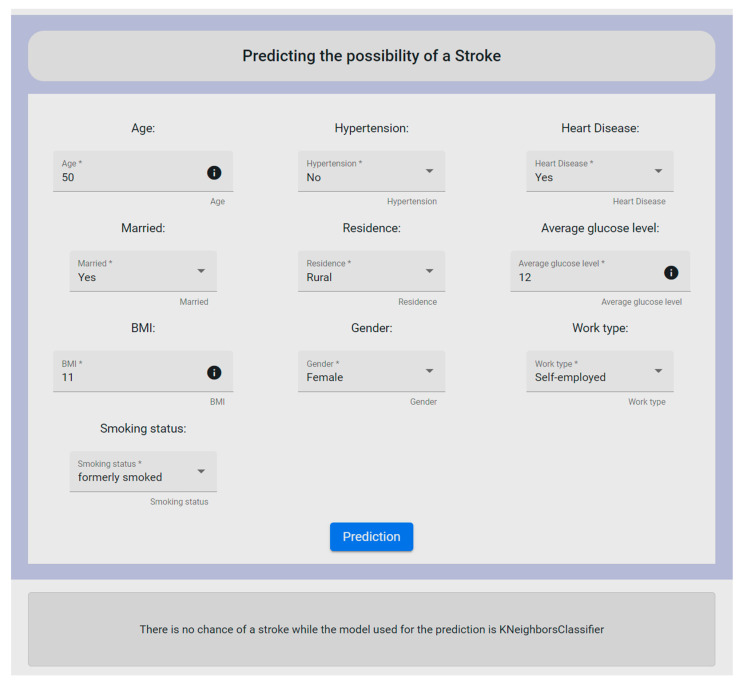
Example of stroke probability form.

To perform a prediction on a given patient, the ML algorithms were already trained with the datasets described in Section 3.1. As a result, for a new prediction based on the trained models, specific information for this patient is uploaded through one of the forms provided by the UI of the mechanism, as shown in Figure 10. More specifically, the form depicted in Figure 10 deals with the stroke dataset, where the user is requested to fill in the provided fields in order to proceed with the needed data analysis. To this end, it should be noted that all form’s fields are required to be filled in by the user. 

The fields that make up the form refer to the id, gender, age, hypertension, heart_disease, ever_married, work_type, residence_type, avg_glucose_level, bmi, and smoking_status. By clicking the prediction button, the user could see whether the patient had a chance of having a stroke or not, based on the provided data. Firstly, the data were cleaned to remove outliers or other similar cases such as missing values of some fields, and then the data were properly orchestrated through the execution of the Orchestrated Experiment microservice. Consequently, the ML models were trained by exploiting the inserted data via the Model Training microservice and then exporting the appropriate metrics via the Model Evaluation and Model Validation microservices. After completing this procedure, to check the performance of the developed models on both the training data and the test data, the Model Serving microservice was applied for the prediction results. At the end, all these mechanisms were optimized into one page within the UI. Additionally, along with the final decision, the UI revealed to the user the most suitable ML model used for the prediction. In this experiment, the mechanism revealed that there was not any possibility of the patient to have a stroke, whereas the KNN algorithm was outlined to have produced the most reliable and efficient results. To be more specific, KNN was chosen for the stroke case since it produced the highest accuracy rate (96%), and also in relation to the remaining metric functions (further analyzed in Table 15). 

Following the same concept, the mechanism supports the same functionalities for the other five datasets (i.e., diabetes, heart failure, COVID-19, breast cancer, kidney disease). In deeper detail, as for the case of diabetes, the form’s features were: age, admission_type, discharge_type, timeHospital, admission_source_type, num_medication_type, num_inpatient_type, number_diagnoses, num_procedure, race, gender, glu_serum, A1C, and change. For the heart failure prediction, the form’s features were age, anaemia, diabetes, ejection_fraction, creatinine_phosphokinase, serum_creatinine, sex, high_blood_pressure, platelets, smoking, serum_sodium, and time. In addition, the features for the COVID-19 case were: age, hematocrit, hemoglobin, platelets, redBloodCells, lymphocytes, leukocytes, basophils, eosinophils, monocytes, serumGlucose, neutrophils, creatinine, urea, sodium, proteina, potassium, alanineTransaminase, and aspartateTransaminase. Furthermore, in the case of breast cancer, the form’s feature were: radius_mean, texture_mean, perimeter_mean, area_mean, smoothness_mean, compactness_mean, concavity_mean, concave points_mean, symmetry_mean, fractal_dimension_mean, radius_se, texture_se, perimeter_se, area_se, smoothness_se, compactness_se, concavity_se, concave points_se, symmetry_se, fractal_dimension_se, radius_worst, texture_worst, perimeter_worst, area_worst, smoothness_worst, compactness_worst, concavity_worst, concave points_worst, symmetry_worst, and fractal_dimension_worst. Finally, for the kidney disease scenario, the form’s features were sg, al, sc, pcv, and htn.

To effectively capture all the aforementioned results for all the different chosen scenarios, as stated above, the proposed mechanism applied the procedure described in Section 2.2, following the sequence of the described processes (i.e., microservices). Hence, during the Data Reliability process, various corrective actions took place upon the diverse datasets, resulting into the results of Table 7. Each row of this table showcases the data inconsistencies that were traced and fixed per dataset, to secure that the performance of the ML algorithms applied in the following step would be the best possible, since it is highly correlated to the quality of the given data.

Then, each ML algorithm was applied upon the inserted user’s data and the respective cleaned dataset. More specifically, the exploited algorithms had been set based on the parameters that are depicted in the following tables (Table 8, Table 9, Table 10, Table 11, Table 12, Table 13 and Table 14), for each different algorithm. Initially, the models’ training began with each algorithm’s parameters being initialized to random values or zeros. An optimization technique was then used to change the initial values as training/learning continued (e.g., gradient descent). The learning method constantly updated the parameter values as learning progressed, whilst the model’s hyperparameter values stayed static.

Table 15 depicts the prediction results of each algorithm in combination with its percentage of accuracy (%). The values in the first sub-field (inside parenthesis) for the cases of heart failure, stroke, COVID-19, and kidney disease refer that there is a possibility of the anomaly to happen (Yes as Y) or not (No as N). In the case of diabetes, it states if there is a need for the patient to uptake insulin (Yes as Y) or if there is a need to uptake insulin along with other medicine (No as N). As for the case of breast cancer, it states if there is a chance of the cancer type being malignant (as M) or being benign (as B). For example, in the case of the first conducted experiment (i.e., stroke), the result indicated that there was not any chance of the patient to have a stroke (N) and the accuracy of the prediction of KNN was 96%. Even though the rest of the algorithms had also high levels of accuracy, as for example the BNB that had an accuracy of 95%, such a small difference in the world of ML is quite important, since in real-world scenarios, such differences are of crucial importance. Additionally, in the case of breast cancer, the results of most algorithms indicated that a large percentage of patient samples have benign breast cancer, so it is not considered as a problem of great concern. This is positive, because it is observed that the LR algorithm scores an accuracy rate equal to 100%, outlining that the patients were dealing with a benign breast cancer. However, this was also proven with additional related parameters that are mentioned in the following sections.

In sequel, to conclude to the best suitable training algorithm for the prediction, during the training phase, the mechanism considered the metric of accuracy along with the metrics of precision, recall, F1-score, and confusion matrix (described in Section 2.2). 

**Figure 11:**
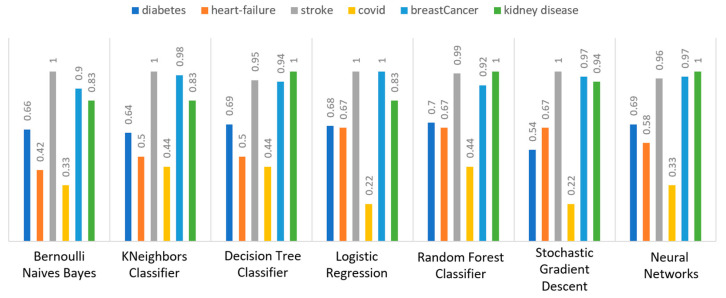
Precision results of ML models for each use case.

Concerning precision, this metric refers to the ratio between the true positive and all the positive values. Thus, for the first experiment (i.e., stroke), this referred to the measure of the patients correctly identified of having a chance to have a stroke from all the patients who actually had. Figure 11 depicts this metric for all the algorithms applied upon the different chosen datasets, where in the case of diabetes the best algorithm was RF (70%), in the case of heart failure both LR, RF, and SGD produced the best results with 67% precision, in the case of stroke BNB, KNN, LR, and SGD had perfect precision (i.e., 100%), whilst in the case of COVID-19 the best performing algorithms were KNN and RF, with 44% precision. For the case of breast cancer, the best algorithm was LR (100%). Finally, as for the kidney disease use case, the best algorithms were DT, RF, and NN, which resulted into 100% precision.

**Figure 12:**
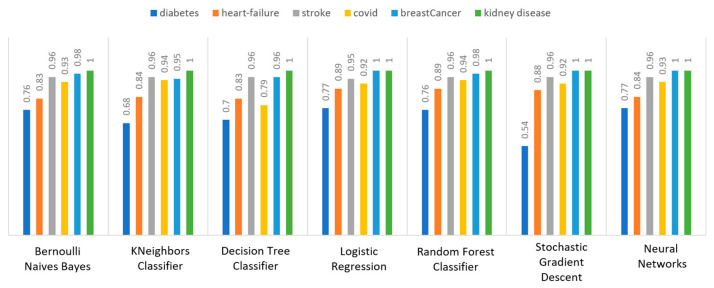
Recall results of ML models for each use case.

Regarding recall, this metric depicts the correctly identified true positive values. Figure 12 depicts this metric for all the algorithms applied upon the different chosen datasets. In the case of diabetes, the best algorithms were LR and NN (77%). When applied on the heart failure dataset both LR and RF had 89% recall, while in the case of the stroke dataset all algorithms had a very high percentage of recall (96%). In the case of COVID-19 the best performing algorithms were KNN and NN with 100% recall, whereas in the same notion, in the case of breast cancer the best algorithms were LR and NN with 100% recall as well. Finally, in the case of kidney disease, DT, RF, and NN also had a perfect recall (100%).

**Figure 13:**
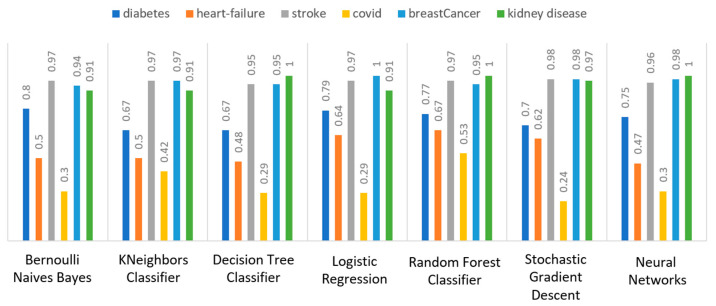
F1-score results of ML models for each use case.

To this end, it should be noted that achieving high recall can be considered more important than obtaining high accuracy. For other kind of ML models, such as the classification ones, whether a patient is suffering from an anomaly or not, it is desirable to have high accuracy. Therefore, Figure 13 portrays the F1-score metric for all the algorithms applied upon all the different selected datasets. In the case of the diabetes dataset, the best algorithm was BNB with 80% F1-score. In the case of heart failure RF had 67% F1-score, while in the case of the stroke dataset BNB, KNN, LR, and RF achieved 97% F1-score. Regarding the COVID-19 dataset, the best algorithm was RF with 53% F1-score. Moreover, in the case of breast cancer dataset LR had 100% F1-score, whilst in the case of the kidney disease dataset DT, RF and NN achieved 100% F1-score as well. It is worth mentioning that SGD algorithm achieved equally accurate results with 97% F1-score.

**Figure 14:**
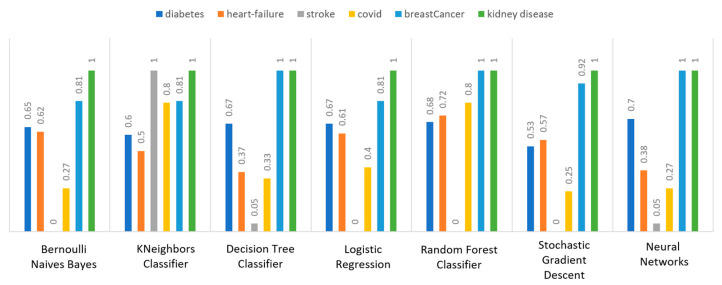
Specificity results of ML models for each use case.

Additionally, regarding the specificity metric, in the scope of the utilized datasets, it refers to the percentage of people who do not have a disease and are tested as negative. Figure 14 depicts this metric for all the algorithms applied upon the different chosen datasets, where in the case of the diabetes dataset the best algorithm was NN (70%), while in the case of the heart failure dataset RF achieved 72% specificity. In the case of the stroke dataset the best choice of algorithm was KNN, since it had 100% recall, whilst in the case of the COVID-19 dataset the best performing algorithms were KNN and RF with 80% specificity. Moreover, regarding the case of breast cancer dataset, the best algorithms were DT, RF, and NN with a perfect (100%) specificity, whereas in the case of the kidney disease dataset all the algorithms achieved a perfect (100%) specificity as well.

#### 3.3.1. Diabetes Use Case 

**Figure 15:**
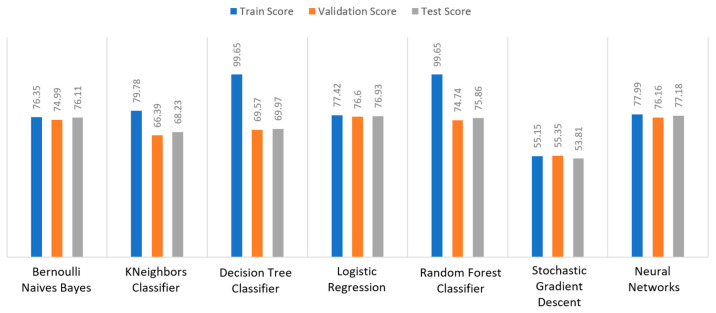
Train–validation–test score for diabetes use case.

Apart from the abovementioned metrics, the train, validation, and test scores were used for the models’ evaluation, as described in Section 2.2. For the diabetes dataset, in Figure 15, DT and RF achieved a train score percentage equal to 99.65%, while the rest of the algorithms achieved a score under 80%. Moreover, it was observed that BNB (76.35%) or KNN (79.78%) could not be properly trained, due to the training data. As for the validation score, only three algorithms achieved a score above 60%. These algorithms were BNB, KNN, and NN, with a percentage equal to 61.22%. Regarding the test score, RF achieved a score of 86%, while all the other algorithms had scores under 85%. The next highest score was 82% by LR. In conclusion, for the diabetes dataset, the best algorithm was BNB, since all the metrics in all training, validation, and test data did not differ from each other.

**Figure 16:**
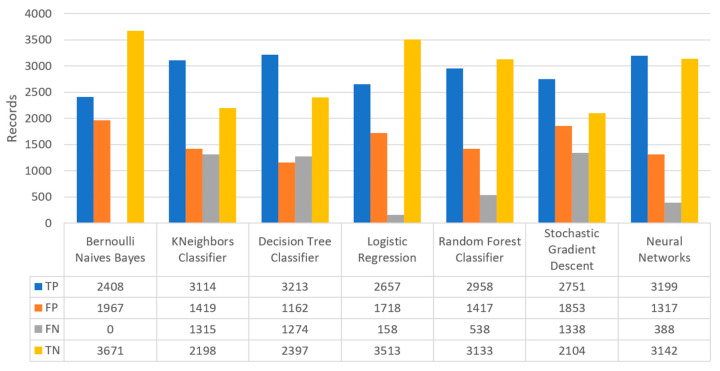
Confusion matrix of prediction results for diabetes use case.

Finally, the confusion matrix for the diabetes scenario’s predictions was estimated, showing the distribution of records based on the four different combinations of predicted and actual values of the diabetes dataset. Figure 16 depicts the confusion matrix for the diabetes dataset, where NN predicted that there was a true probability that insulin would be granted in 3199 records (TP), while insulin in combination with some other medicine would be granted in 3142 records (TN). In addition, the next best algorithm was LR with 2657 records in which insulin would be administered, and 3513 records in which insulin with other medicine would be administered.

**Figure 17:**
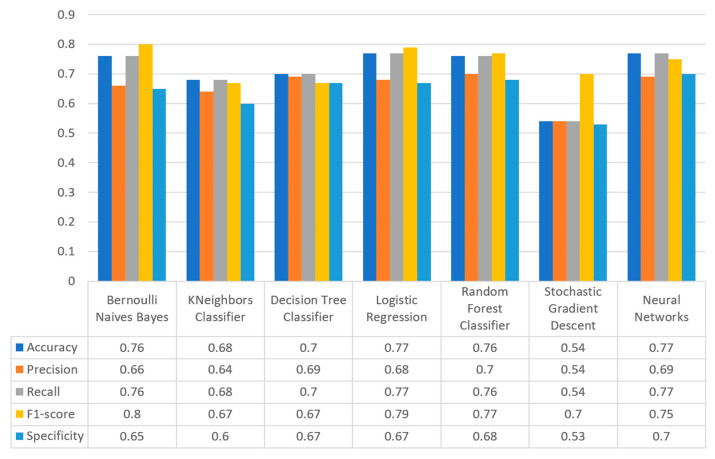
Performance comparison in the diabetes use case.

Figure 17 summarizes the comparison in terms of accuracy, precision, recall, F1-score, and specificity for the diabetes dataset. It can be observed that LR and NN produced the best prediction in terms of accuracy in comparison with BNB and the rest of the algorithms. Furthermore, the highest value of precision was observed for RF, which was equal to 0.7, followed by LR and NN with values equal to 0.68 and 0.69, respectively. In addition, as for the recall metric, the highest value was noted for the LR and NN algorithms. For the F1 score it was observed that the highest value was that of BNB, which was equal to 0.8, while LR, RF, and NN were the next ones. Finally, another important metric was that of specificity in which it is observed that the highest value was noted for NN algorithm, being equal to 0.7. Therefore, it is understood that for this case the most suitable algorithm for the diabetes scenario was NN.

#### 3.3.2. Stroke Use Case

**Figure 18:**
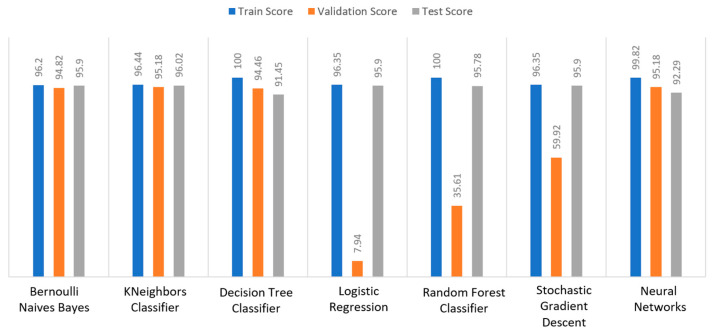
Train–validation–test score for stroke use case.

Regarding the stroke dataset, as shown in Figure 18, KNN achieved a validation percentage equal to 95.18%, BNB 94.82%, and DT 94.46%, while the worst results were those of LR (7.94%) and RF (35.61%), due to the fact that they could not properly validate the received data. Additionally, it is observed that DT and RF, by considering a percentage of data from 65%–70% of the original (training) dataset, achieved a perfect (100%) score, while KNN achieved 96.44%. Additionally, most algorithms produced quite high test scores, however KNN achieved a percentage equal to 96.02%, followed by BNB with a percentage of 95.9%.

**Figure 19:**
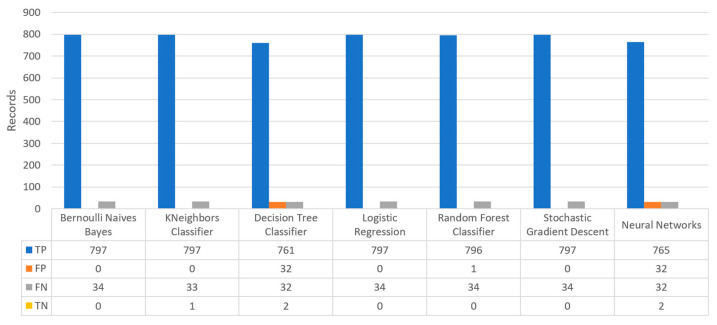
Confusion matrix of prediction results for stroke use case.

Figure 19 depicts the confusion matrix for the stroke dataset, where KNN predicted that there was a true probability that a stroke attack would occur in 797 records (TP), while in only one case (just in 1 record) it predicted that no stroke attack would occur (TN).

**Figure 20:**
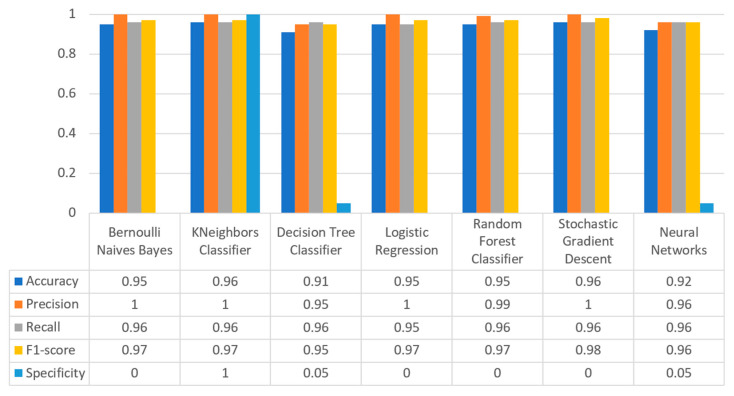
Performance comparison in the stroke use case.

Figure 20 summarizes the comparison in the metrics of accuracy, precision, recall, F1-score, and specificity for the stroke scenario. To begin with, it can be seen that KNN and RF produced the best predictions in terms of accuracy in comparison with BNB, LR, and the rest of the algorithms. Moreover, the precision’s highest value was noted for BNB, KNN, LR, and SGD, which was equal to 1, followed by the RF algorithm. Regarding the recall metric, the highest value was noted in KNN. Moreover, regarding F1-scores, the highest value was achieved by SGD that was equal to 0.98, followed by BNB, KNN, LR, and RF. Finally, another important metric was that of specificity, in which it was observed that the highest value was noted for KNN, being equal to 1. Therefore, it is understood that for this case the most suitable algorithm for predicting whether a patient is likely to have a stroke or not was KNN.

#### 3.3.3. Heart Failure Use Case 

**Figure 21:**
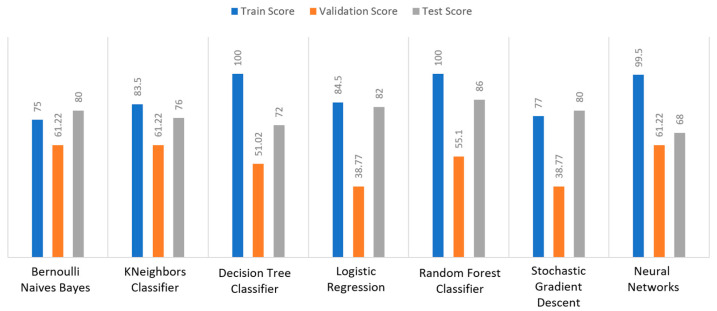
Train–validation–test score for heart failure use case.

For the heart failure dataset, as shown in Figure 21, DT and RF achieved a train score percentage equal to 100%, RF had also a good performance (99.50%), whilst the rest of the algorithms achieved scores between 75% and 85%. Moreover, the lowest percentages were produced by BNB (75%), KNN (83.5%), and SGD (85.32%), due to the fact that the training data could not be properly trained. Regarding the second metric, validation scores were quite low for all the algorithms, whilst only three of them achieved a score above 60%, referring to BNB, KNN, and NN. As for the test score, RF achieved a score equal to 86%, while all the other algorithms had scores below 85%, while the next highest percentage was achieved by LR (82%). In conclusion, for the case the of heart failure dataset, the best algorithm was BNB, since all the captured metrics in all the training, validation, and test data did not differ from each other.

**Figure 22:**
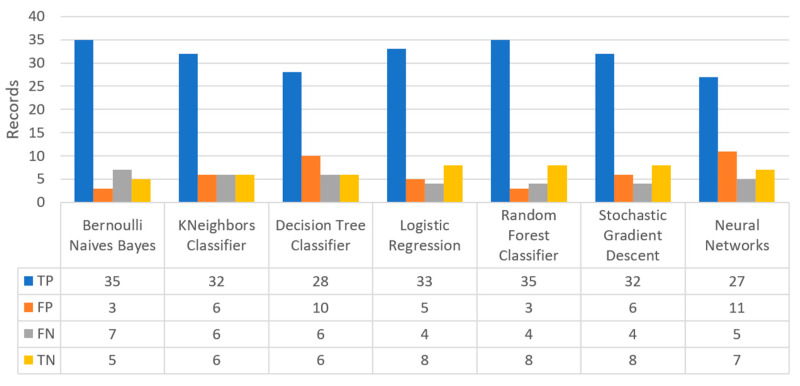
Confusion matrix of prediction results for heart failure use case.

Figure 22 shows the confusion matrix for the heart failure dataset, where RF predicted that there was a true probability that a heart failure attack would occur in 35 records (TP), while no heart failure attack would occur in eight records (TN). Additionally, the next best algorithm was LR with 33 records being predicted for having a heart failure attack, while no heart failure attack was predicted in eight records.

**Figure 23:**
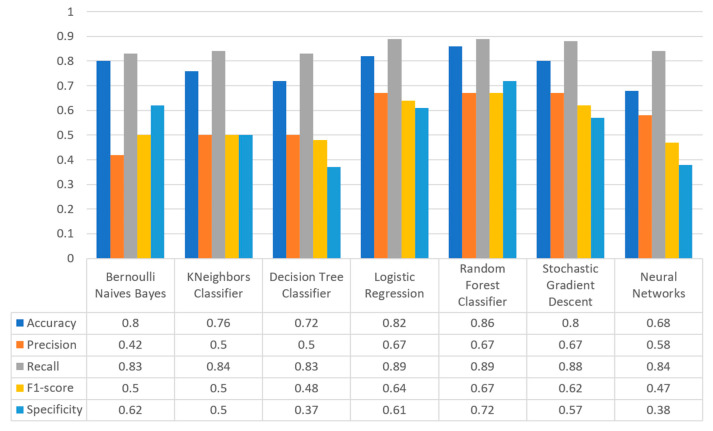
Performance comparison in the heart failure use case.

Figure 23 illustrates the comparison for the different metrics in the heart failure case. RF produced the best prediction in terms of accuracy (0.86) in comparison with LR (0.82) and the other algorithms. In addition, regarding precision, it is observed that the highest value was noted for RF and LR that was equal to 0.67. As for the recall metric, the highest value was noted for the LR and RF algorithms, while for F1-score, it is observed that the highest value was that of RF, being equal to 0.67, and followed by LR and SGD (<0.04). Finally, another important captured metric was that of specificity, in which it is observed that the highest value was achieved by RF, being equal to 0.72. Therefore, it is understood that the most suitable prediction regarding heart failure was produced by RF.

#### 3.3.4. COVID-19 Use Case 

**Figure 24:**
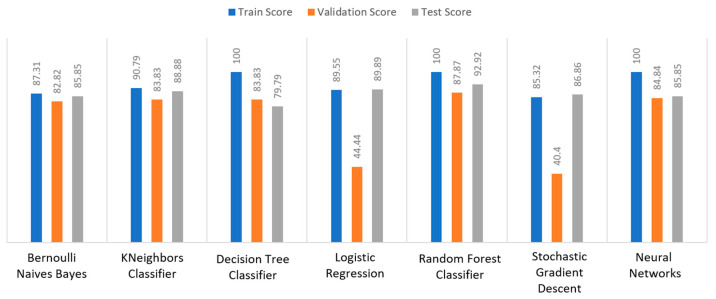
Train–validation–test score for COVID-19 use case.

For the COVID-19 dataset, in Figure 24 it can be seen that DT and NN achieved a train score percentage equal to 100% and KNN achieved a 90.79% score, while the rest of the algorithms reached a score not greater than 90%. For example, BNB had an 87.31% score, LR had an 89.55% score, and SGD had an 85.32% score due to the fact that the training data could not be properly trained. It was also observed that most algorithms achieved a score of around 80 to 90% (i.e., BNB (82.82%) or RF (87.87%)), while LR and SGD achieved a very low score of around 40% to 45%, respectively. Regarding the test score metric, RF achieved a score of 92.92%, while all the other algorithms had scores under 90% (i.e., BNB (85.85%) or LR (89.89%)). In conclusion, for the case of the COVID-19 dataset, the best algorithm was RF, because all the metrics in all the training, validation, and test data achieved a maximum score of approximately or equally to 100%.

**Figure 25:**
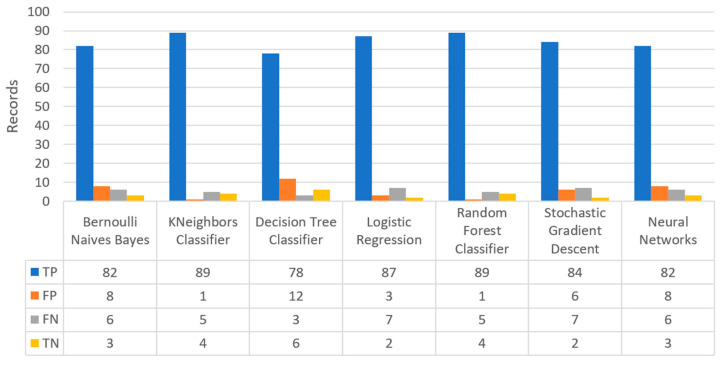
Confusion matrix of prediction results for COVID-19 use case.

Figure 25 shows the confusion matrix for the COVID-19 dataset, where KNN predicted that there was a true probability that a pneumonia attack would occur in 89 records (TP), while no pneumonia attack would occur in just four records (TN). However, it is worth mentioning that RF also depicted the same results.

**Figure 26:**
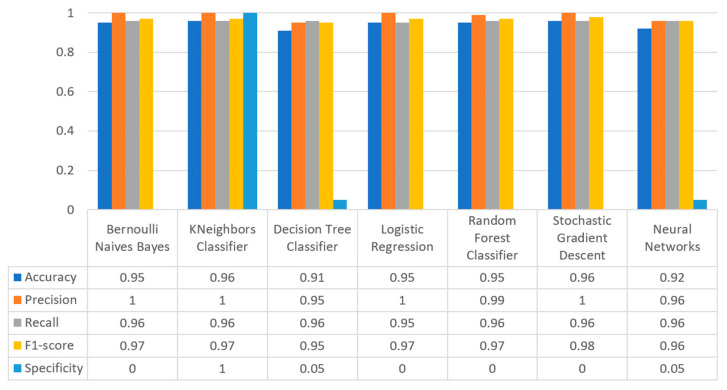
Performance comparison in the COVID-19 use case.

Figure 26 summarizes the comparison for all the captured metrics of COVID-19 dataset. As it can be seen, KNN produced the best prediction in terms of accuracy (0.96) in comparison with LR (0.95) and the other algorithms. In addition, precision’s highest value was noted for BNB, KNN, LR, and SGD, which was equal to 1. Moreover, as for the recall metric, the highest value was noted for BNB and KNN (0.96). For F1-score it was observed that the highest value was that of SGD that was equal to 0.98. Finally, for the specificity metric, it was observed that the highest value was captured for KNN, being equal to 1. Therefore, it is understood that for this case, the most suitable prediction for having a pneumonia or not was the KNN algorithm.

#### 3.3.5. Breast Cancer Use Case 

**Figure 27:**
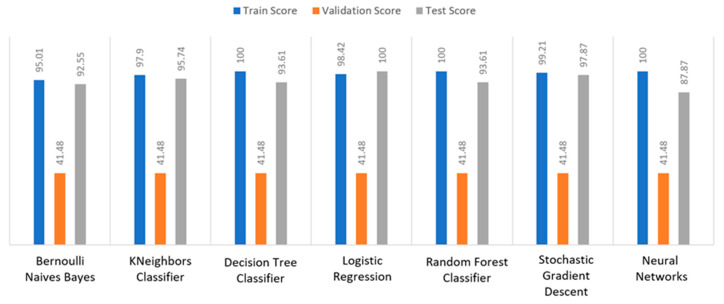
Train–validation–test score for breast cancer use case.

For the breast cancer scenario, in Figure 27 it is illustrated that all the algorithms achieved an almost perfect train score percentage between 95% to 100%. For example, DT, RF and NN had a percentage equal to 100%, while the other algorithms achieved more than 95% (i.e., BNB (95.01%) or KNN (97.9%)). However, regarding the validation score, all the algorithms achieved a low percentage at 41.48%. As for the test score, LR achieved 100% score, whereas the second better algorithm was SGD with 97.87%. In addition, the next better algorithm was KNN with 95.74%. All the other algorithms had scores below 95%. Thus, it appeared that the best algorithm was LR, since the minimum deviation was observed between the training and test metrics.

**Figure 28:**
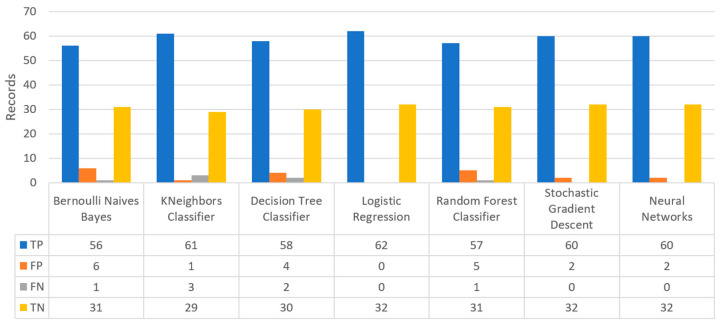
Confusion matrix of prediction results for breast cancer use case.

Figure 28 presents the confusion matrix for the breast cancer dataset, where LR predicted that there was a true probability that benign cancer would be in 62 records (TP), while malignant cancer would be in 32 records (TN). Moreover, the next best two algorithms were SGD and NN, which predicted that 60 records would have benign cancer, while 32 records would have malignant cancer.

**Figure 29:**
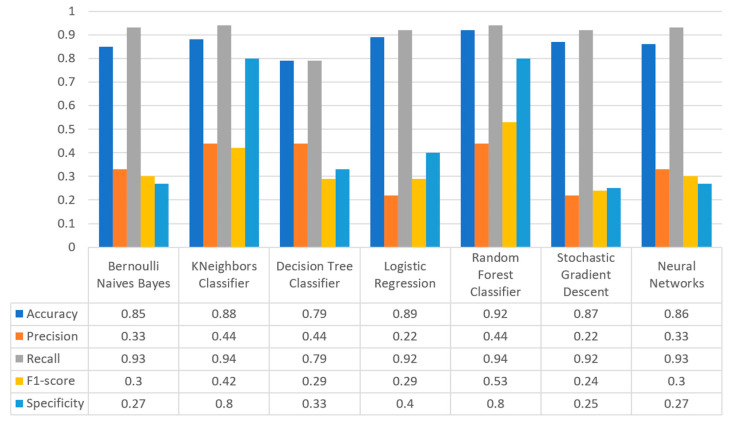
Performance comparison in the breast cancer use case.

Figure 29 synopsizes the comparison in the captured metrics of accuracy, precision, recall, F1-score, and specificity for the breast cancer case. More specifically, RF produced the best prediction in terms of accuracy (0.92) in comparison with LR (0.89) and KNN (0.88). Moreover, precision’s highest value was noted for RF which was equal to 0.44. As for the recall metric, the highest value was noted for the RF and KNN (0.94), whilst for F1-score it is observed that the highest value was that of algorithm RF, being equal to 0.53. Finally, as for specificity, it was observed that the highest value was noted for RF, being equal to 0.8. Therefore, it is understood that for this case the most suitable algorithm regarding the prediction of whether the patient is likely to have a benign or malignant cancer for the breast cancer was RF.

#### 3.3.6. Kidney Disease Use Case 

**Figure 30:**
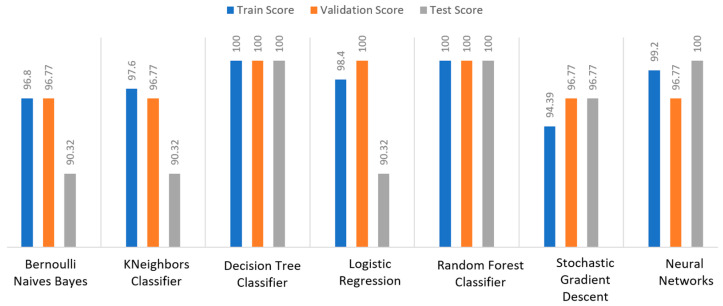
Train–validation–test score for kidney disease use case.

For the kidney disease dataset, as it is depicted in Figure 30, DT and RF achieved a train score percentage equal to 100%, while NN scored an equally high percentage equal to 99.2%. However, it should be emphasized that the scores of the rest of the algorithms were also quite high (90%). Regarding the validation score, DT, LR, and RF achieved a tremendously high score (100%), whilst the other algorithms achieved quite high scores as well, equal to 96.77%. Regarding the test score metric, DT, RF, and NN achieved an equal score at 100%, whilst the next highest score was that of SGD (96.77%). In conclusion, for the kidney disease dataset, the best algorithms were DT and RF, since all the metrics in all the training, validation, and testing data did not differ from each other, being equal to 100%.

**Figure 31:**
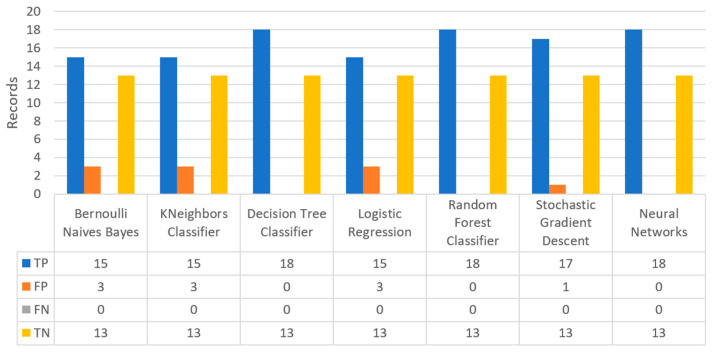
Confusion matrix of prediction results for kidney disease use case.

Figure 31 shows the confusion matrix for the kidney disease dataset, where DT, RF and NN predicted that there was a true probability that 18 records would refer to patients with kidney disease (TP), while 13 records would refer to patients with no kidney disease (TN).

**Figure 32:**
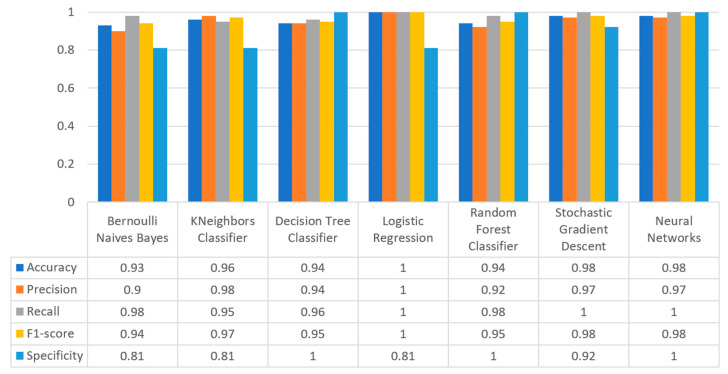
Performance comparison in the kidney disease use case.

Figure 32 summarizes the comparison for all the captured metrics of the kidney disease case. In deeper detail, LR produced the best prediction in terms of accuracy (1) in comparison with LR (0.93). In addition, precision’s highest value was noted for the algorithm LR, being equal to 1, recall achieved the highest value by the LR, SGD, and NN algorithms (1), whilst F1-score had the highest value by LR, being equal to 1. Finally, regarding the specificity metric, it is observed that the highest value was achieved by the DT, RF, and NN algorithms, being equal to 1. Therefore, it is understood that in this case, the most suitable algorithm regarding the prediction of kidney disease was LR.

#### 3.3.7. Training Performance 

**Figure 33:**
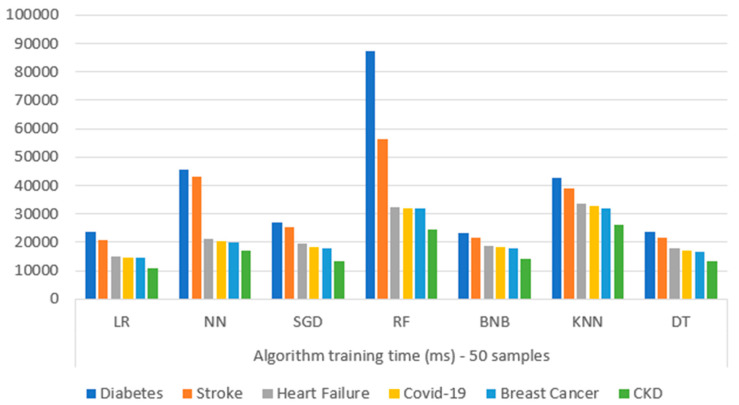
Training performance comparison for each algorithm per dataset.

Figure 33 presents the comparison of the training performance time (in milliseconds (ms)) for each ML algorithm upon each different chosen dataset. Specifically, for each dataset, consecutive tests were performed on the Model Training microservice, implementing the different exploited ML algorithms (BNB, KNN, DT, LR, RF, NN, and SGD). Based on the captured results, it is observed that RF was more complex in its training process for the diabetes dataset, while NN followed. In general, it can be seen that the diabetes dataset, due to its high complexity (concerning its large number of features, and huge data volume), revealed the highest training time in every algorithm. As for the rest of the datasets, it can be observed that the training time was a function of data complexity, as expected, where RF took longer to train its model, while NN and KNN followed.

## 4. Discussion

Applying ML techniques and algorithms in several diverse domains is a matter of investigation in a plethora of research and enterprise initiatives. ML provides the right tools to analyze data and extract useful knowledge. More specifically, regarding the healthcare domain, ML is capable of processing large amounts of data and then providing useful insights regarding the planning and the delivering of care by the clinicians [101]. ML can lead to better decision-making, thus minimizing the cost whilst, at the same time, maximizing the efficiency and efficacy of healthcare-related processes [102].

It is an undeniable fact that a variety of research approaches have been proposed, presenting and applying diverse ML algorithms, even combining those algorithms for achieving high rates of predictions’ data accuracy [103], focusing on performing predictions on multidimensional heterogeneous health-related data for inference in medical practices. As previously stated, all of these current methodologies have been effectively used in medical research for the construction of prediction models, leading in undoubtedly effective and correct decision-making. A summarization of various conducted research works upon diverse healthcare-related use case datasets can be found in Table 16. It should be noted that the illustrated healthcare-related research works refer to the diverse healthcare scenarios (i.e., stroke, COVID-19, diabetes, breast cancer, kidney disease, heart failures) that are under investigation in the current manuscript.

Regarding the aforementioned list of existing research works, it is also worth mentioning the corresponding key components that are part of every approach’s workflow towards the accomplishment of the required predictions (except for the ML algorithms that they utilize, as depicted in Table 16). Through this analysis, it becomes feasible to determine the works’ applicability and complexity in comparison with the manuscript’s proposed mechanism, based on the separate components that both of them put in place. Table 17 depicts in deep detail such information, including a list of the existing approaches in comparison with the proposed mechanism. To be more specific, the components listed in this table refer to “Gateway”, “Data Reliability”, “Hyperparameters’ Tuning” (included into the Model Training component), “Data Storage”, and “Model Evaluation” that are considered to be the major contributions of the current manuscript (as described in Section 1). It is worth mentioning that regarding the first two components (i.e., “Gateway”, “Data Reliability”), those do not refer to simple data collection and data cleaning techniques (e.g., data are stored into a simple local file and are cleaned by dropping rows of erroneous data), since those are trivial procedures. Instead, they refer to approaches that the corresponding components perform more complex tasks, such as those that are showcased in the present manuscript (further analyzed in Section 2.2). Moreover, it should be noted that the “Data Storage” component does not refer to the fact whether the existing research works exploit a NoSQL database for storing their data (as in the case of the proposed mechanism), but it just refers to the fact as to whether the stated research works are putting in place in their overall workflow a Data Storage component for handling the storage of the investigated data. 

Based on the results captured in Table 16 and Table 17, it has become clear that even though new and better software technologies have considerably reduced the complexity of implementation for many ML algorithms in recent years, most of these approaches are use-case specific, whereas specific algorithms and components have been applied upon the effective completion of their predictions. For this reason, this manuscript proposes a mechanism that utilizes a list of widely used and well-established ML algorithms to train models to perform predictions across diverse healthcare anomalies’ scenarios, and based on specific metrics, it compares the algorithms’ efficiency. By the time that this process gets complete, the mechanism creates a catalogue with the most proper algorithms to be applied on each given scenario. The outcomes of this process are further described below, regarding each different metric that was estimated across the diverse chosen scenarios.

More specifically, regarding the diabetes use case, it is observed that the most suitable algorithm, in terms of the accuracy parameter, is LR with a percentage equal to 77%, given that the number of correct predictions (insulin administration) divided by the total number of predictions are correctly defined. Furthermore, as for the heart failure scenario, it is observed that the most proper algorithm is RF with an accuracy equal to 86. Moreover, in the stroke use case scenario, the most efficient algorithm is SGD with an accuracy percentage of 96%. As for COVID-19 use case scenario, RF performed most sufficiently since it achieved the highest accuracy of 92%. Additionally, on finding breast cancer, the results indicate that LR had the best performance with 92%, referring to all the cases in which the patients will have benign cancer, while for the kidney disease case, it seems that this will not happen in the given patients, with 100% accuracy of the three algorithms of DT, RF, and NN representing a strong prediction.

Additionally, the recall metric is considered quite significant because it shows the ratio of correctly predicted positive observations to all observations in the actual class. For all the use cases studied in this manuscript it is observed that the recall metric is above 50%. In the diabetes use case, the highest corresponding score is equal to 77% and is achieved by the LR algorithm. In the heart failure use case, a percentage of 89% is achieved by LR and RF. In the rest of the use cases, it appears that recall scores are greater than 90%, except for the use case of COVID-19, where the recall score is 79%.

Regarding the precision metric and the diabetes use case, it is observed that the most suitable algorithm is RF with a rate equal to 70%, given the number of correct predictions (insulin administration) divided by the total defined number of predictions. Furthermore, in the heart failure use case, it is observed that the most suitable algorithms were LR, RF, and SGD, with a precision score of 67%. Additionally, in the stroke use case, the most effective algorithms were BNB, KNN, LR, and SGD, with a perfect precision score equal to 100%. As for the COVID-19 use case, RF had the most adequate performance as it achieved the highest precision score of 44%. Additionally, for the breast cancer use case, the mechanism shows that LR was quite precise (100%), while the rest of the algorithms range between 90% and 100%, showing that the given patients will have benign cancer, while in the case of kidney disease, it seems that there will not be any kidney disease to the patients with 100% precision for three algorithms (DT, RF, NN), which is a strong prognostic factor.

Additionally, regarding the F1-score metric, the weighted average of precision and recall was tested. Initially, in the diabetes use case, it appears that BNB adapts better with a percentage of 80%, followed by LR with a score equal to 79%. In the heart failure use case, it is observed that RF’s F1-score is 67%. However, in general the algorithms have a quite low score regarding F1-score (around 50%). In the same way, in the use case of COVID-19, it seems that only RF surpasses 50%, whilst for the remaining scenarios (i.e., stroke, breast cancer, and kidney disease), all the algorithms appear to match well with percentages greater than 90%. For example, in the stroke case it appears that SGD has a percentage equal to 98%, in the case of breast cancer LR achieves a score equal to 100%, while in the same notion, in the kidney disease case it appears that several algorithms (DT, RF, NN) achieve a perfect score (100%) as well.

Of course, it is worth mentioning that the current approach has certain limitations. Regarding the Gateway microservice, this utilizes a mechanism to efficiently retrieve large amount of data by splitting them into batches and storing them into the database. However, the size of the batches is currently at a default value. If the batch size was dynamically changing based on the size and structure of the collected data, the data collection would probably be even more efficient. Moreover, this functionality has been tested with a limited number of external sources and third-party APIs, so further testing should take place. As for the Data Reliability microservice, this depends on a set of ML techniques that even include NLP. This fact suggests that more time and/or computational cost should be needed in order to effectively eradicate all the possible data inconsistencies. As a result, further development should take place to make the corresponding processes more efficient, at a software level. Moreover, the Data Reliability microservice should split the data to batches to perform data cleaning, when it comes to large datasets. As for the microservices of Model Training, Model Evaluation, and Model Validation, it is difficult to compare algorithms objectively across studies, since each study’s performance is reported using different methodologies on different populations with distinct sample distributions and features. To be fair, algorithms must be compared using the same independent test set that is representative of the target population and the same performance criteria. Without this, healthcare practitioners would have a difficult time determining which algorithm is most likely to perform well for their patients. To fully examine the performance of the different available algorithms in a representative sample of their community, each healthcare practitioner may use the curation of separate local test sets. Such independent test sets should be constructed using an unenriched representative sample and data that are not intended to be used to train algorithms. Furthermore, prior to formal testing, an additional local training dataset for the Model Serving microservice could be provided to allow fine tuning of the chosen algorithms. ML algorithms are susceptible to a variety of flaws, including inapplicability outside of the training domain, bias, and brittleness (i.e., the ability to be easily deceived [138]). The following have to be considered: the dataset shift, the fitting confounders rather than the true signal, the spreading inadvertent biases in clinical practice, the offering of algorithm interpretability, the creation of correct evaluations of model confidence, and the difficulty of applicability to new populations. Given the current velocity of innovation, the significant risks involved, and the potentially fluid nature of ML models, this is a one-of-a-kind problem. Furthermore, proactive regulation will create trust in professionals and healthcare systems for the Prediction microservice. What is more, currently there is a trade-off between the performance and the explainability for the developed Performance Monitor microservice. The highest performing models (e.g., DL) are frequently the least explainable, whereas models with worse performance (e.g., LR, DT, or RF) are the most explainable. DL models currently have a significant disadvantage in the way that they lack explicit declarative knowledge representation, making it difficult to provide the necessary explanatory structures [139]. For the Orchestrated Experiment microservice, the currently exploited ML models do not rely on a long history of research in classical symbolic AI techniques to allow for the encoding of data semantics and the use of ontologies to assist the learning process, which may help healthcare specialists to better understand and re-trace decision processes [140]. Finally, regarding the developed UI, all the underlying microservices are directly or indirectly connected and visualize their produced results through the provided interfaces, thus following the MSA towards code’s reuse and efficient operations. However, in the case of adding more complex ML/DL mechanisms, more complex code compositions are required that should be further studied to respond to the requests returned to the developed UI.

## 5. Conclusions

Currently there is a growing interest in using ML to predict the outcome of a disease or treatment, while there exists a plethora of algorithms that can be utilized for this task. However, each algorithm is efficient for specific kind of data and use cases. Thus, the selection of the most proper algorithm is not an easy task. This manuscript proposes a mechanism that utilizes well-known ML algorithms, and conducts a complete comparison between them, resulting with the most effective ones in different scenarios being related to healthcare. The reason behind this is to construct a catalogue of ready-to-use and on-demand ML algorithms, and to offer—under specific criteria—the most appropriate algorithms for more efficient and reliable prediction results.

According to the conducted study, the most accurate algorithms for diabetes are LR with a percentage of 77% accuracy and RF with an accuracy of 86%. For heart failure, it is observed that the most proper algorithm is RF with an accuracy of 86%. For stroke, SGD is the most efficient algorithm, with an accuracy percentage of 96%. As for COVID-19, RF achieved an accuracy of 92%. In the breast cancer use case LR achieved an accuracy rate equal to 92%, while for kidney disease, DT, RF, and NN achieved 100% accuracy. Regarding recall, it is a measure indicating how well the algorithm predictions match the actual observations, where in the current study, it was found that this metric was always above 50%. The highest score was achieved by LR in the diabetes use case, with a score of 77%. In the heart failure use case, RF achieved a score of 89%, while the rest of the use cases had recall scores greater than 90%. In the COVID-19 use case, the same algorithm had a recall score of 79%. As for the precision metric, in the diabetes use case, it was found that RF performed the best, with a rate equal to 70%. In the heart failure use case, it was found that LR, RF, and SGD had the best precision scores, with 67%, 100%, and 100%, respectively. In the stroke use case, BNB, KNN, LR, and SGD had the best precision scores (100%), whilst for the COVID-19 use case, it was found that RF had the best performance, with a precision score of 44%. In the breast cancer use case, it was found that LR had the best performance, with a precision score of 100%. In general, it appeared that the algorithms had low scores when it comes to the F1-score metric. However, in a few specific cases, the algorithms were better suited for certain tasks, as for example in the case of heart failure prediction, where RF was better suited for predicting the occurrence of heart failure, while in the case of stroke, SGD was better for predicting the occurrence of stroke.

The results of this study are particularly useful in assisting the selection of classification algorithms for future applications that exploit relevant health-related data. As for the next steps, among the first actions that should be considered are the address of the limitations already discussed in the discussion section. We have already identified potential solutions and workarounds to these limitations, and as a result our future goals include the resolution of these challenges. In addition, we aim to address issues related to feature importance regarding the overall training of the ML algorithms, considering additional hyperparameters tuning research studies. Moreover, we plan to take into consideration the AUC metric for capturing the algorithms’ performance, as well as to perform several experiments in cloud computing premises by utilizing different kinds of architectural styles [141]. We also envision utilizing datasets from other domains to investigate whether certain ML algorithms are more efficient than others, based on the type of the datasets’ features. Finally, we aim to test the current ML algorithms in distributed environments and datasets by utilizing state-of-the-art techniques such as Federated Learning (FL).

## Figures and Tables

**Table 1:** Dataset description of diabetes.

No.	Attribute Name	Attribute Information	Range of Values
1	Race	Race of patient	“Caucasian”, “Asian”, “African”, “American”, “Hispanic”, “Other”
2	Gender	Gender of patient	“Male”, “Female”, “Unknown/Invalid”
3	Age	Age of patient	(0–10), …, (90,100)
4	Admission type	Type of admission	(1–8)
5	Discharge disposition	Disposition of discharge	(1–28)
6	Admission source	Source of admission	(1–20)
7	Time in hospital	Number of days between admission and discharge	(1–14)
8	Number of procedures	Number of operations conducted during the encounter	(0–6)
9	Number of medications	Number of different names used throughout the encounter	(1–81)
10	Number of inpatient visits	Number of inpatient visits in the year preceding the encounter	(0–21)
11	Number of diagnoses	Number of diagnoses that have been entered into the system	(1–16)
12	Glucose serum test result	Range of result/Test not taken	“>200”, “>300”, “Normal”, “None”
13	A1c test result	Range of result/Test not taken	“>8”, “>7”, “Normal”, “None”
14	Change of medications	Change in diabetic medications (either dosage or generic name)	“Change”, “No change”

**Table 2:** Dataset description of stroke.

No.	Attribute Name	Attribute Information	Range of Values
1	id	Unique identifier	(67–72.940)
2	gender	Patient’s gender	“Male”, “Female”, “Other”
3	age	Patient’s age	(0.08–82)
4	hypertension	Patient has hypertension or not	(0–1) 0: Does not have hypertension, 1: Has hypertension
5	heart_disease	Patient has heart_disease or not	(0–1) 0: Does not have any heart diseases, 1: Has heart diseases
6	ever_married	Patient is or not married	“No”, “Yes”
7	work_type	Type of work type	“Children”, “Govt_jov”, “Private” “Never_worked”,“Self-employed”
8	residence_type	Residence type of patient	“Rural”, “Urban”
9	avg_glucose_level	Average blood glucose level	(55.12–271.74)
10	bmi	Body mass index	(10.3–97.6)
11	smoking_status	Patient smoking status	“Formerly smoked”, “Never smoked”, “Smokes”, “Unknown”

**Table 3:** Dataset description of heart failure.

No.	Attribute Name	Attribute Information	Range of Values
1	age	Age of the patient	(40–95)
2	anemia	Decrease of red blood cells or hemoglobin	(0–1) 0: red blood cells, 1: hemoglobin
3	creatinine_phosphokinase	Blood’s CPK enzyme (mcg/L)	(23–7861)
4	diabetes	If the patient has diabetes	(0–1) 0: patient has diabetes, 1: patient has not diabetes
5	ejection_fraction	Percentage of blood that leaves the heart with each contraction	(14–80)
6	high_blood_pressure	If the patient has hypertension	(0–1) 0: patient has hypertension, 1: patient has not hypertension
7	platelets	Platelets found in the blood (kiloplatelets/mL)	(25,100–850,000)
8	serum_creatinine	Blood’s serum creatinine (mg/dL)	(0.5–9.4)
9	serum_sodium	Blood’s serum sodium (mEq/L)	(113–148)
10	sex	Woman or man	(0–1) 0: woman, 1: man
11	smoking	Whether or not the patient smokes	(0–1) 0: patient smokes, 1: patient does not smoke
12	time	Follow-up period (days)	(4–285)
13	death_event	If the patient deceased during the follow-up period	(0–1) 0: patient has deceased during the follow-up period, 1: patient has not deceased during the follow-up period

**Table 4:** Dataset description of COVID-19.

No.	Attribute Name	Attribute Information	Range of Values
1	Patient age quantile	Age of the patient	(0–19)
2	Hematocrit	Quantity of hematocrit	(−4.50–2.66)
3	Hemoglobin	Quantity of hemoglobin	(−4.34–2.67)
4	Platelets	Quantity of platelets	(−2.55–9.53)
5	Red blood Cells	Quantity of red blood cells	(−3.97–3.64)
6	Lymphocytes	Quantity of lymphocytes	(−1.86–3.76)
7	Leukocytes	Quantity of leukocytes	(−2.02–4.52)
8	Basophils	Quantity of basophils	(−1.14–11.07)
9	Eosinophils	Quantity of eosinophils	(−0.83–8.35)
10	Monocytes	Quantity of monocytes	(−2.16–4.53)
11	Serum Glucose	Quantity of serum glucose	(−1.10–7.00)
12	Neutrophils	Quantity of neutrophils	(3.33–2.53)
13	Urea	Quantity of urea	(−1.63–11.24)
14	Proteina C reativa mg/dL	Quantity of proteina C reativa	(−0.53–8.02)
15	Creatinine	Quantity of creatinine	(−2.38–5.05)
16	Potassium	Quantity of potassium	(−2.28–3.40)
17	Sodium	Quantity of sodium	(−5.24–4.09)
18	Alanine transaminase	Quantity of alanine transaminase	(−0.64–7.93)
19	Aspartate transaminase	Quantity of aspartate transaminase	(−0.70–7.23)

**Table 5:** Dataset description of breast cancer.

No.	Attribute Name	Attribute Information	Range of Values
1	radius_mean	Radius of lobes	(6.98–28.1)
2	texture_mean	Mean of surface texture	(9.71–39.3)
3	perimeter_mean	Outer perimeter of lobes	(43.8–189)
4	area_mean	Mean area of lobes	(144–2501)
5	smoothness_mean	Mean of smoothness levels	(0.05–0.16)
6	compactness_mean	Mean of compactness	(0.02–0.35)
7	concavity_mean	Mean of concavity	(0–0.43)
8	concave points_mean	Mean of concave points	(0–0.2)
9	symmetry_mean	Mean of symmetry	(0.11–0.3)
10	fractal_dimension_mean	Mean of fractal dimension	(0.05–0.1)
11	radius_se	SE of radius	(0.11–2.87)
12	texture_se	SE of texture	(0.36–4.88)
13	perimeter_se	Perimeter of SE	(0.76–22)
14	area_se	Are of SE	(6.8–542)
15	smoothness_se	SE of smoothness	(0–0.03)
16	compactness_se	SE of compactness	(0–0.14)
17	concavity_se	SEE of concavity	(0–0.4)
18	concave points_se	SE of concave points	(0–0.05)
19	symmetry_se	SE of symmetry	(0.01–0.08)
20	fractal_dimension_se	SE of fractal dimension	(0–0.03)
21	radius_worst	Worst radius	(7.93–36)
22	texture_worst	Worst texture	(12–49.5)
23	perimeter_worst	Worst perimeter	(50.4–251)
24	area_worst	Worst area	(185–4250)
25	smoothness_worst	Worst smoothness	(0.07–0.22)
26	compactness_worst	Worse compactness	(0.03–1.06)
27	concavity_worst	Worst concavity	(0–1.25)
28	concave points_worst	Worst concave Points	(0–0.29)
29	symmetry_worst	Worst symmetry	(0.16–0.66)
30	fractal_dimension_worst	Worst fractal dimension	(0.06–0.21)

**Table 6:** Dataset description of kidney disease.

No.	Attribute Name	Attribute Information	Range of Values
1	Id	Unique ID	(1–399)
2	Age	Age of the patient	(2–90)
3	Bp	Blood pressure	(50–180)
4	Sg	Specific gravity	(1–1.02)
5	Al	Albumin	(0–5)
6	Su	Sugar	(0–5)
7	Rbc	Red blood cells	“Normal”, “Unknown”
8	Pc	Pus cell	“Normal”, “Unknown”
9	Pcc	Pus cell clumps	“Not present”, “Present”, “Unknown”
10	Ba	Bacteria	“Not present”, “Present”, “Unknown”
11	Bgr	Blood glucose random	(70–490)
12	Bu	Blood urea	(10–391)
13	Sc	Serum creatinine	(0.4–76)
14	Sod	Sodium	(4.5–163)
15	Pot	Potassium	(2.7–47)
16	Hemo	Hemoglobin	(3.1–17.8)
17	Pcv	Packed cell volume	(9–54)
18	Wc	White blood cell count	(0–9600)
19	Rc	Red blood cell count	(0–4.5)
20	Htn	Hypertension	”True”, ”False”
21	Dm	Diabetes mellitus	”No”, ”Yes”, ”Other”
22	Cad	Coronary artery disease	”No”, ”Yes”, ”Other”
23	Appet	Appetite	”Good”, ”Poor”
24	Pe	Pedal edema	”True”, ”False”
25	Ane	Anemia	”True”, ”False”

**Table 7:** Cleaning results for each use case.

Dataset	Number of Records	Cleaning Metrics		
		Missing Values	Outliers Values	Duplicate Values
Diabetes	101,766	100,723	0	0
Stroke	5110	201	0	1
Heart Failure	299	0	19	0
COVID-19	600	0	0	0
Breast Cancer	569	0	5	0
Kidney Disease	400	684	0	0

**Table 8:** Parameters of mechanism set for BNB.

Parameter	Set Value	Description
alpha	1.0	Additive parameter (Laplace/Lidstone) used for smoothing
binarize	0.0	Threshold used for mapping to booleans a sample feature
fit_prior	True	Learn class prior probabilities
class_prior	None	Prior probabilities of the classes

**Table 9:** Parameters of mechanism set for KNN.

Parameter	Set Value	Description
n_neighbors	5	Integer number corresponding to the neighbors
weights	Uniform	All the points in each neighborhood are equally weighted
algorithm	Auto	The most proper algorithm is chosen based on the values that are passed to the fit method
Leaf_size	30	Leaf size that is passed to BallTree or KDTree
metric	2	Minkowski distance (equivalent to standard Euclidean metric)

**Table 10:** Parameters of mechanism set for DT.

Parameter	Set Value	Description
criterion	Gini	Function to review the quality of a split
splitter	Best	Strategy to choose the splitting method at each node
max_depth	None	Maximum depth of tree (if None, nodes are expanded until all leaves are pure or contain less than min_samples_split)

**Table 11:** Parameters of mechanism set for RF.

Parameter	Set Value	Description
n_estimators	100	Integer number corresponding to the trees in the forest
criterion	Gini	Function to review a split
max_depth	None	Maximum depth of the tree
min_samples_split	2	Integer number that minimizes the number of samples required to split an internal node
min_samples_leaf	1	Integer number that minimizes the number of samples required to be at a leaf node
min_weight_fraction_leaf	0.0	Minimum weighted fraction of the total of weights (of all the input samples) required to be at a leaf node
max_features	sqrt	Number of features to consider when looking for the best split
max_leaf_nodes	None	Grow trees with max_leaf_nodes in best-first fashion, where best nodes are defined as relative reduction in impurity
min_impurity_decrease	0.0	A node will be split if this split induces a decrease of the impurity greater than or equal to this value
bootstrap	True	Use bootstrap samples when building trees
oob_score	False	Use out-of-bag samples to estimate the generalization score
n_jobs	None	Number of jobs to run in parallel
random_state	None	Randomness of samples’ bootstrapping when building trees and sampling of features when looking for the best node’s split
verbose	0	Verbosity when fitting and predicting
warm_start	False	Fit a whole new forest
class_weight	None	Weight of each class
ccp_alpha	0.0	Parameter used for Minimal Cost-Complexity Pruning
max_samples	None	Draw X.shape [0] samples

**Table 12:** Parameters of mechanism set for LR.

Parameter	Set Value	Description
solver	liblinear	Algorithm to use in the optimization problem
penalty	l2	Additive penalty term (L2)
dual	True	Dual or primal formulation (Dual formulation is only implemented for L2 penalty with liblinear solver)
tol	10^-4^	Tolerance for stopping criteria
C	1.0	Inverse of regularization strength, where smaller values specify stronger regularization
fit_intercept	True	A constant should be added to the decision function
intercept_scaling	1	Used for solver ‘liblinear’ self.fit_intercept ‘True’
class_weight	None	No class weigh
random state	None	Shuffle data
max_iter	100	Maximum number of iterations for solvers to converge
multi_class	Auto	Selects ‘ovr’ if data is binary or if solver = ‘liblinear’, otherwise selects ‘multinomial’
verbose	0	Verbosity level

**Table 13:** Parameters of mechanism set for ANN.

Parameter	Set Value	Description
hidden_layer_sizes	5000, 10	Represents the number of neurons in the *i*th hidden layer
activation	relu	Activation function for the hidden layer
solver	lbfgs	Optimizer in the family of quasi-Newton methods
alpha	10^-5^	Strength of the L2 regularization term
batch_size	Auto	Size of minibatches for stochastic optimizers
learning_rate	Constant	Learning rate schedule for weight updates
learning_rate_init	0.001	Initial learning rate for step-size in updating the weights
power_t	0.5	Exponent for inverse scaling learning rate
max_iter	200	Maximum number of iterations
shuffle	True	Whether to shuffle samples in each iteration
random_state	None	Random number generation for weights and bias initialization
tol	10^-4^	Tolerance for the optimization
verbose	False	Print progress messages to stdout
warm_start	False	Erase the previous solution
momentum	0.9	Momentum for gradient descent update
nesterovs_momentum	True	Use Nesterov’s momentum
early_stopping	False	Use early stopping to terminate training when validation score is not improving
validation_fraction	0.1	Proportion of training data to set aside as validation set for early stopping
max_fun	15000	Maximum number of loss function calls

**Table 14:** Parameters of mechanism set for SGD.

Parameter	Set Value	Description
loss	hinge	Loss function
penalty	l2	Penalty (regularization) to be used
alpha	0.0001	Constant that multiplies the regularization term
fit_intercept	True	Intercept should be estimated
max_iter	5000	Maximum number of passes over training data (epochs)
tol	10^-3^	Stopping criterion
shuffle	True	Training data should be shuffled after each epoch
verbose	0	Verbosity level
epsilon	0.1	Epsilon in the epsilon-insensitive loss functions
n_jobs	None	Number of CPUs for One Versus All (OVA) computation
random_state	None	Shuffling the data
learning_rate	optimal	eta = 1.0///(alpha * (t + t0)) where t0 is chosen by a heuristic
power_t	0.5	Exponent for inverse scaling learning rate
early_stopping	False	Use of early stopping to terminate training when validation score is not improving
validation_fraction	0.1	Proportion of training data to set aside as validation set for early stopping
n_iter_no_change	5	Number of iterations with no improvement to wait before stopping fitting
class_weight	None	Preset for the class_weight fit parameter
warm_start	False	Erase the previous solution

**Table 15:** Prediction results and accuracy per ML model for each use case.

Dataset	ML Algorithms						
	BNB	KNN	DT	LR	RF	SGD	NN
Diabetes	Y(76%)	N(68%)	Y(70%)	Y(77%)	Y(76%)	N(54%)	N(77%)
Stroke	N(95%)	N(96%)	Y(91%)	Y(95%)	N(95%)	N(96%)	N(92%)
Heart Failure	Y(80%)	N(76%)	N(72%)	Y(82%)	Y(86%)	N(80%)	N(68%)
COVID-19	N(85%)	N(88%)	N(79%)	N(89%)	N(92%)	N(87%)	N(86%)
Breast Cancer	B(93%)	B(96%)	B(94%)	B(100%)	B(94%)	B(98%)	B(98%)
Kidney Disease	N(90%)	N(90%)	N(100%)	N(90%)	N(100%)	N(97%)	N(100%)

**Table 16:** List of existing research works based on diseases and algorithms (annotated with *) used for their predictions.

Disease	Author	Methods						
		BNB	KNN	NN	SGD	DT	LR	RF
Diabetes	Mogaveera et al. (2021) [104]					*		
	Wu et al. (2022) [105]						*	
	Xing et al. (2007) [106]			*		*		
	Oza et al. (2022) [107]		*				*	
	Palimkar et al. (2022) [108]	*				*	*	*
	Komal et al. (2019) [109]							*
COVID-19	Ahmad et al. (2018) [110]	*						
	Ho et al. (2022) [111]				*			
	Oyelade et al. (2021) [112]				*			
	Hassan Yaseen et al. (2022) [113]		*					*
	Shaban et al. (2020) [114]		*					
	Yoo et al. (2020) [115]					*		
Heart Failure	Akbulut et al. (2018) [116]	*				*	*	*
	Peter et al. (2012) [117]	*	*	*		*		
	Morgenstern et al. (2022) [118]							*
	Qian et al. (2022) [119]						*	*
	Çınar et al. (2021) [120]			*				
Stroke	Ponciano-Rodríguez et al. (2019) [121]						*	
	Santos et al. (2022) [122]					*		
	Dev et al. (2022) [123]			*			*	*
	Paikaray et al. (2022) [124]		*					*
	Iosa et al. (2021) [125]			*				
Kidney Disease	Pal et al. (2022) [126]					*	*	
	Revathy et al. (2022) [127]						*	*
	Sinha et al. (2015) [128]		*					
	Almustafa et al. (2015) [129]	*	*		*			
	Singh et al. (2022) [130]			*				
	Kim et al. (2021) [131]			*				
Breast Cancer	Mittal et al. (2015) [132]				*			
	Tran et al. (2022) [133]			*				*
	Pfob et al. (2022) [134]			*		*		
	Rasool et al. (2022) [135]		*				*	
	Naseem et al. (2022) [136]		*	*		*	*	
	Allugunti et al. (2022) [137]			*				*

**Table 17:** List of existing research works based on the components (annotated with *) used for their predictions.

Author	Components				
	Gateway	Data Reliability	Hyperparameters Tuning	Data Storage	Model Evaluation
Proposed Mechanism	*	*	*	*	*
Mogaveera et al. (2021) [104]				*	*
Wu et al. (2022) [105]				*	*
Xing et al. (2007) [106]				*	*
Oza et al. (2022) [107]			*		*
Palimkar et al. (2022) [108]					*
Komal et al. (2019) [109]				*	*
Ahmad et al. (2018) [110]					*
Ho et al. (2022) [111]			*	*	*
Oyelade et al. (2021) [112]		*			*
Hassan Yaseen et al. (2022) [113]			*		*
Shaban et al. (2020) [114]		*		*	*
Yoo et al. (2020) [115]					*
Akbulut et al. (2018) [116]	*			*	*
Peter et al. (2012) [117]					*
Morgenstern et al. (2022) [118]					*
Qian et al. (2022) [119]	*			*	*
Çınar et al. (2021) [120]			*		*
Ponciano-Rodríguez et al. (2019) [121]					*
Santos et al. (2022) [122]			*		*
Dev et al. (2022) [123]			*		*
Paikaray et al. (2022) [124]					*
Iosa et al. (2021) [125]					*
Pal et al. (2022) [126]			*		*
Revathy et al. (2022) [127]					*
Sinha et al. (2015) [128]					*
Almustafa et al. (2015) [129]					*
Singh et al. (2022) [130]		*	*		*
Kim et al. (2021) [131]			*		*
Mittal et al. (2015) [132]			*		*
Tran et al. (2022) [133]					*
Pfob et al. (2022) [134]			*		*
Rasool et al. (2022) [135]			*		*
Naseem et al. (2022) [136]			*		*
Allugunti et al. (2022) [137]			*		*

## Data Availability

Not applicable.
